# Boron-rich benzene and pyrene derivatives for the detection of thermal neutrons

**DOI:** 10.1038/srep13401

**Published:** 2015-09-03

**Authors:** Henok A. Yemam, Adam Mahl, Unsal Koldemir, Tyler Remedes, Sean Parkin, Uwe Greife, Alan Sellinger

**Affiliations:** 1 Department of Chemistry and Geochemistry, Colorado School of Mines, Golden, CO 80401, USA; 2 Department of Physics, Colorado School of Mines, Golden, CO 80401, USA; 3 Department of Chemistry, University of Kentucky, Lexington, KY 40506, USA

## Abstract

A synthetic methodology is developed to generate boron rich aromatic small molecules based on benzene and pyrene moieties for the detection of thermal neutrons. The prepared aromatic compounds have a relatively high boron content up to 7.4 wt%, which is important for application in neutron detection as ^10^B (20% of natural abundance boron) has a large neutron induced reaction cross-section. This is demonstrated by preparing blends of the synthesized molecules with fluorescent dopants in poly(vinyltoluene) matrices resulting in comparable scintillation light output and neutron capture as state-of-the art commercial scintillators, but with the advantage of much lower cost. The boron-rich benzene and pyrene derivatives are prepared by Suzuki conditions using both microwave and traditional heating, affording yields of 40–93%. This new procedure is simple and straightforward, and has the potential to be scaled up.

Plastic scintillators are polymer-based detector materials for gamma radiation, fast neutrons and other charged particles^1^,^2^,^3^. Their low cost, fast-timing resolution and ease of large-scale production make it a first-line detection method compared to inorganic crystal scintillators^4^,^5^. However, due to the absence of high neutron capture isotopes in plastic scintillators, they are unable to detect thermal neutrons and are therefore concurrently used with ^3^He gas detectors at international borders and airports to detect illicit trafficking of special nuclear materials (SNM)^6^. Due to ^3^He scarcity and increasing demand, alternative isotopes such as ^10^B and ^6^Li with comparable thermal neutron capture cross sections and higher natural abundances have been investigated^7^,^8^. Current developments of neutron sensitive plastic scintillators mainly rely on commercially available carboranes as a boron source due to their high boron content^9^. Thermal neutrons are detected via the capture reaction on the nucleus of ^10^B and measuring the scintillation light produced by the alpha particles (^4^He) released by this reaction, shown in equation (1)^10^. Although carboranes have high boron content (~75% wt.), they have limited solubility in plastic scintillator formulations, are very expensive, and the cost is significantly higher in their ^10^B enriched form.


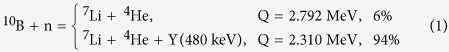


Alternative methods of thermal neutron detection include boron containing semiconductor crystals, enriched boron-10 fluoride (^10^BF_3_) gas filled proportional counters, and boron lined tube counters along with liquid scintillators doped with boron compounds such as trimethyl borate^11^,^12^,^13^. However, growing crystals in large quantities for significant area coverage is difficult and ^10^BF_3_ has severe limitations in deployment due to its toxicity^14^,^15^. While boron lined tubes are physically similar to ^3^He tubes, they suffer from reduced efficiencies due to the energy loss effects from having a solid boron wall coverage. Trimethyl borate mixed into liquid scintillators of many varieties has a very low flash point and is required to be very well sealed from oxygen in order to reduce quenching effects and maintain efficiency. Other isotopic candidates for scintillators such as ^6^Li or ^155^Gd/^157^Gd are not as attractive as ^10^B due to higher cost, lack of availability, and reduced compatibility with inexpensive polymer matrices^16^,^17^,^18^,^19^. Furthermore, the price of ^10^B containing additives to these matrices needs to be comparable to that of the polymers in order to achieve neutron sensitivity in a cost effective manner. Alternatives to carboranes need to be produced with efficient synthesis methods and inexpensive reagents.

With regard to boron containing organic materials, recently direct borylation of activated C–H bonds of aromatic compounds has been reported using iridium-based catalysis^20^,^21^,^22^,^23^,^24^,^25^. However, high Ir catalyst loadings, lack of regioselectivity and longer reaction times hinder its applicability and scale up potential. In order to counter these disadvantages, the synthesis of 1,3,6,8-tetrakis(4,4,5,5-tetramethyl-1,3,2-dioxaborolan-2-yl)pyrene was reported by Yamada and coworkers by nickel catalyzed direct borylation achieving a yield of 74% in two days^26^. Furthermore, synthesis of 1,2,4,5-tetrakis(4,4,5,5-tetramethyl-1,3,2-dioxaborolan-2-yl)benzene was reported by Wagner and coworkers with an overall yield of 64%; however, their synthetic process was a two-step reaction system achieving only partial borylation and the use of highly pyrophoric and toxic reagents such as n-butyllithium and Grignard reagents^27^. Both Aubert *et al.* and Gandon *et al.* utilized cobalt-catalyzed [2+2+2] cycloaddition of ethynyl pinacol borate to yield a mixture of 2,2′,2″-(benzene-1,2,4-triyl)tris(4,4,5,5-tetramethyl-1,3,2-dioxaborolane) and 1,3,5-tris(4,4,5,5-tetramethyl-1,3,2-dioxaborolan-2-yl)benzene with a yield of 63%^28^,^29^. Their use of an expensive borylating reagent (ethynyl pinacol borate—$650/g) and a difficult separation of the product mixture could be detrimental to using this reaction system. Compared to cobalt-catalyzed cycloaddition reactions, Wang *et al.* achieved 85% yield by direct borylation of 1,3,5-tribromobenzene using Miyuara conditions^30^,^31^.

We report here the borylation of multi-halo functionalized benzene and pyrene derivatives using the very efficient and mature Suzuki chemistry to afford soluble materials with boron content as high as 7.43 wt%. Furthermore some of these materials also have strong blue luminescence properties that may contribute to scintillation efficiency for detecting both gamma and neutron radiation. Examples of polymer-based scintillators using our new materials demonstrate highly efficient scintillation and thermal neutron detection.

## Results and Discussion

### Synthesis

**Figure 1:**
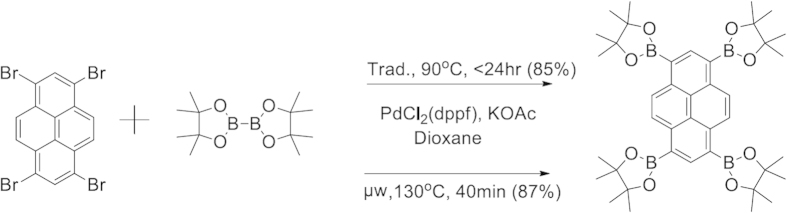
Conventional heating condition (Trad.) and Microwave (μW) heating condition for generating tetra-borylated pyrene (TBP). These conditions were used for all the reactions.

We have applied traditional and microwave assisted Suzuki conditions to promote the borylation of bromo functionalized aromatics using commercially available and cost effective bispinacolato diborane (B_2_Pin_2_)^32^. In our efforts to complete these reactions within a reasonable time, we used slight excess equivalents of B_2_Pin_2_ to complete the multiple borylations. Increasing the heating to 90 °C was crucial for completing these reactions in less than 24 hours (Fig. 1) and our microwave assisted conditions resulted in similar reaction yields in much shorter reaction times (40 min vs. 24 hr).

**Figure 2:**
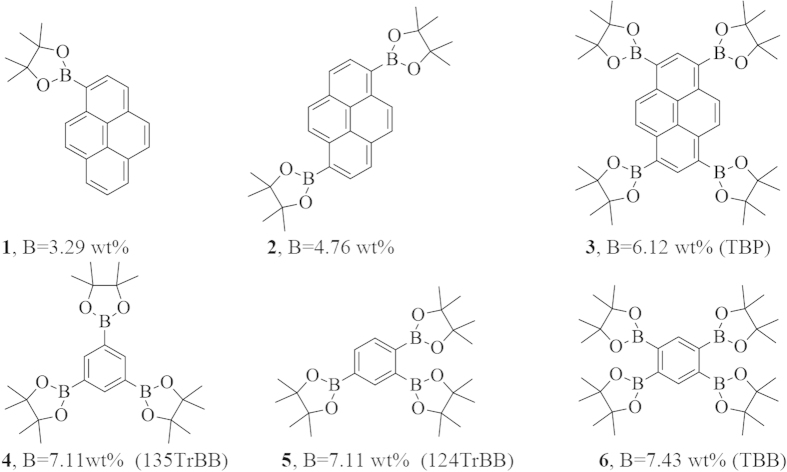
Boron containing pyrene and benzene derivatives.

To show applicability of the aforementioned conditions to other aromatic molecules, related boron containing molecules (Fig. 2) were synthesized using lower catalyst loading, shorter reaction times, simpler purification methods, and comparable synthesis yields as previous literature methods^33^.

To our knowledge, use of microwave methods for multiple borylation has only been reported for diborylation, where in our approach we demonstrate multiple borylations (tri and tetra) in a significantly reduced time frame of 40–60 min^34^,^35^. Table 1 summarizes conditions and percent yield comparison between traditional and microwave assisted reactions.

The synthesis of **1** (Fig. 2, entry 1) was previously reported by Akhavan-Tafti *et al.* with a similar procedure to our traditional synthesis (except 85 °C, DMSO) affording approximately the same percent yield (Table 1)^36^. We believe we are the first to report the synthesis of this molecule by microwave-assisted borylation. Both ^1^H and ^13^C NMR for compound **1** are found in Figure S1. Compound **2** synthesis showed the biggest drop in yield when attempting microwave borylation (79% vs 63%). Several attempts were made by varying temperature, amounts of catalyst and B_2_Pin_2_, and reaction time, however the microwave yield couldn’t be improved. Both NMR and MALDI TOF MS for this compound confirm the product purity and can be found in Figures S2 and S6 respectively.

**Figure 3:**
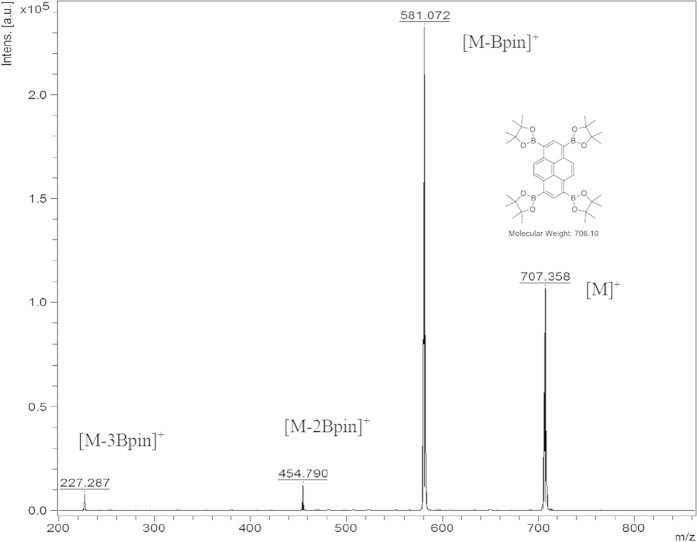
MALDI-TOF-MS of TBP with 1,8,9-trihydroxyanthracene as a matrix.

The synthesis of TBP (Fig. 2, entry 3) appeared straightforward but the characterization was problematic as both ^1^H and ^13^C NMR were inconclusive, resulting in broad and featureless peaks in the aromatic region while showing definitive and clear peaks in the aliphatic region. This was thought to be the result of the presence of a paramagnetic ion or of the large difference between the number of aliphatic and aromatic hydrogens (48:6). As such, many attempts were made to solve this problem by varying deuterated solvents, increasing relaxation time, utilizing chromium(III) acetylacetonate (Cr(acac)_3_) as a relaxing agent, and attempting solid-state NMR^37^. Unfortunately, a conclusive NMR confirming the successful synthesis of TBP couldn’t be produced. Even though this problem was not stated explictly in the literature, we have noticed similar reports confirming our observation^24^. Despite this shortcoming, we turned our attention to analysing this molecule by MALDI TOF MS that confirmed the molecule as shown in Fig. 3. By utilizing 1,8,9-trihydroxyanthracene as a matrix, all the possible fragments 707.4 (M^+^), 581.1, 454.8 and 227.3 Da were observed.

**Figure 4:**
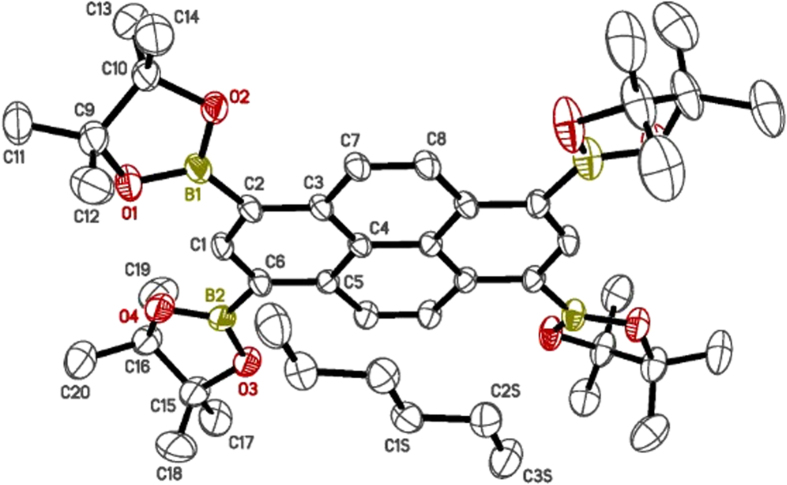
An ellipsoid plot (50% probability) for TBP.

Encouraged by this result, TBP crystals with dimensions of 1–2  mm were prepared by slow introduction of hexanes into a TBP chloroform solution. The crystals had suitable quality for single-crystal x-ray analysis, revealing TBP and n-hexane molecules each sitting on a 2-fold rotation axis as shown in Fig. 4. The pyrene ring system is essentially flat, but the Bpin rings are non-planar and disordered over two distinct conformations^38^,^39^,^40^. The crystal structure of TBP coupled with MALDI shows we have unequivocally synthesized this molecule despite our inability to obtain conclusive ^1^H and ^13^C NMR. More information with regard to the TBP crystal structure can be found in the supplementary information.

The synthesis of **135TrBB** and **124TrBB** (Fig. 2, entry 4 & 5) had significance in determining if the symmetry of boron containing molecules could have an effect in the detection of thermal neutrons, especially because these two molecules have identical amounts of boron by mass (7.11%). NMR for both of these molecules can be found in Figures S3 and S4. As with the TBP molecule, 124TrBB also provided ^1^H NMR spectra with high integration ratios between the aliphatic and aromatic protons. We addressed this issue by running the NMR experiment in d_6_-DMSO (rather than CDCl_3_) at 80 °C (rather than room temperature). Also GC/MS results confirmed the formation and purity of 124TrBB.

Generally, the yield for conventional heating was slightly improved (except for entry **6**) over the microwave approach (Table 1). Entry 6 (Fig. 2, TBB) was helpful in understanding the lack of accurate NMR spectra for TBP since its aliphatic to aromatic proton ratio is higher (48:2 to 48:6). However, both ^1^H and ^13^C NMR unambiguously confirmed the synthesis of this molecule (Figure S5). The crystal structure and two-step reaction synthesis of this molecule was published by Wagner *et al.*^27^. More detailed information on the synthesis and characterization of all the materials can be found in the supplementary information.

## Discussion

Plastic scintillators are a composite of a matrix (PVT) that absorbs radiation energy and transfers this energy mainly to a primary dopant (PPO) via Förster resonance energy transfer (FRET)^41^. The PPO emission is then with nearly 100% efficiency absorbed by a wavelength shifter (POPOP) that has an efficient fluorescence peak matched to the photomultiplier tube (PMT) sensitivity^42^,^43^. Table 2 shows the composition of plastic scintillators prepared incorporating the synthesized boron materials (entry 3–6, Fig. 2) into the PVT matrix. The first eight samples were colorless with intense blue luminescence under UV excitation. As TBP has a pale yellow color, samples ix–xi were optically clear with a yellowish color and strong blue luminescence under UV excitation.

The light output of these samples (4.7 cm diameter × 1.1–1.3 cm thickness) was compared to a commercial scintillator (EJ-204) of approximately the same dimensions prepared by Eljen Technology. Our control (Table 2, sample i) resulted in 95% of the light output compared to the commercial scintillator. A summary of light output of samples i–xi compared to EJ-204 and their properties are shown in Table 3. The high average molecular weight (Mn and Mw) of the samples (as determined by GPC using poly(styrene) calibration standards) is indicative of complete polymerization of the scintillator samples, hence minimal inhibition of polymerization by the added components. As seen in column 5 (Table 3), the signal produced by the 1.48 MeV alpha and 0.48 MeV ^7^Li ion (products of thermal neutron reaction with ^10^B) is quenched to produce scintillation light equivalent in amplitude from an electron with an energy of 60–100 kev depending on sample composition.

Samples ii–iv (Figure S15) contain increasing amounts of 135TrBB (Fig. 2, entry 4). As expected, capture of thermal neutrons was not observed for the 0.5% sample of this compound due to the low concentration of ^10^B (0.007% wt) (Sample ii). Increasing the amount of 135TrBB to 1% showed thermal capture as well as increased light output (sample iii), while increasing the amount to 5% wt reached the solubility limit of 135TrBB in PVT (sample iv) resulting in an opaque sample. We speculate that the symmetrical nature of the compound was contributing to its crystallization in PVT at higher loadings.

**Figure 5:**
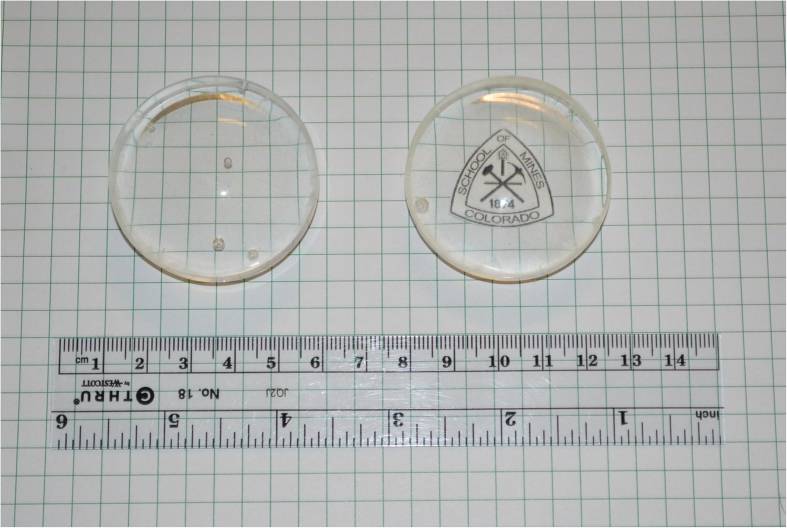
Left to right: Sample v and vi incorporating 1 and 5% 124TrBB respectively.

To address this issue, we prepared and utilized 124TrBB that has a more unsymmetrical structure but the same boron content as 135TrBB. Samples v and vi both had the best optical clarity with 124TrBB (Fig. 2, entry 5) as the boron additive (Fig. 5) indicating enhanced solubility of 124 versus 135TrBB. Increasing the concentration of 124TrBB from 1% to 5% wt increased both the light output as well as thermal neutron capture (Table 3, entry v and vi).

**Figure 6:**
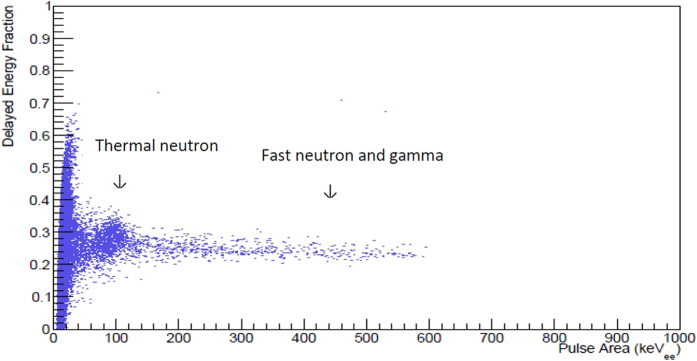
Thermal neutron capture using a 5% 124TrBB plastic scintillator, sample vi.

Figure 6 shows a one minute collection of data using the ^244^Cm/^13^C source for 5% 124TrBB (Table 2, entry vi), that already shows distinct neutron capture above the background noise. This sample resulted in a ^10^B thermal neutron capture signal at approximately 92 keVee with 81% relative light output. To our knowledge, this is the highest thermal neutron capture signal observed from a boron doped plastic scintillator.

Samples vii and viii both had TBB (Fig. 2, entry 6) as boron additive. The solubility limit of this compound was the lowest in PVT. For example, 1% wt loadings showed crystallization as shown in (Figure S16). Despite its poor solubility, it showed a clearly visible boron capture signal even at 1% (Table 3, entry vii). Increasing the concentration to 5% wt decreased the optical clarity significantly as shown in Figure S16. We propose that the decrease in light output compared to our standard was due to attenuation of light by increased dopant concentration. This effect is in agreement with literature reports.

**Figure 7:**
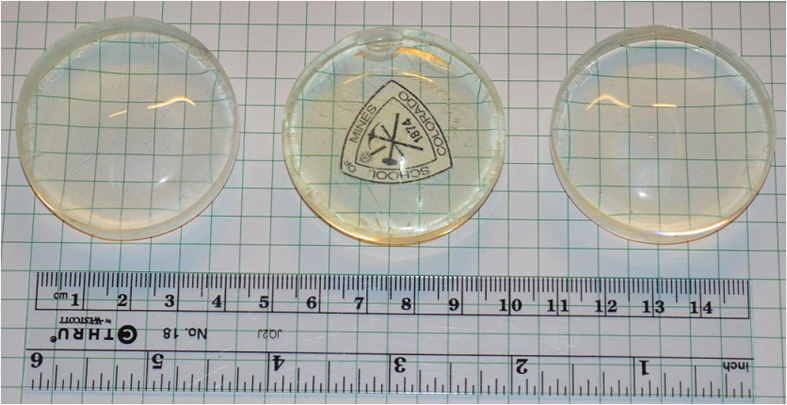
Left to right: Sample ix, x and xi incorporating 1, 2 and 1% TBP respectively.

Utilizing TBP (Fig. 2, entry 3) as both a boron source and primary dopant resulted in lower light output and neutron capture likely due to unoptimized energy transfer from the matrix to TBP and wavelength shifter (Figure S8). The dramatic drop in light output also caused the capture reaction to be buried in the electronic background. The issue was resolved by only utilizing TBP as a boron source and using PPO as the primary dopant (Table 3, entry xi). This sample showed a dramatic increase in light output and the thermal neutron induced reaction signal became clearly visible (Table 3, entry xi). All of the samples containing TBP (Fig. 7) were slightly yellow due to its pale yellow color. Absorption and emission spectra for TBP can be found in Figure S7.

## Conclusion

Both traditional and microwave-assisted synthesis of direct multi-borylation of pyrene and benzene derivatives achieved high percent yields and purity of desired products. The simplicity of these synthetic routes together with inexpensive starting materials and ease of scale up production could be highly advantageous in reducing the cost of boron-rich additives for plastic scintillators. These synthesized boron additives doped with commercially utilized PPO and POPOP fluorescent emitters in poly(vinyltoluene) matrices have demonstrated successful thermal neutron induced reactions with comparable/improved light output compared to commercial samples using very expensive carborane derivatives. In the case of 124 TrBB, the ^10^B neutron capture signal registered a stronger signal than state-of-the-art boron doped plastic scintillators. We are currently working on the synthesis of ^10^B enriched versions of our boron-rich additives in order to increase thermal neutron capture probability. Additionally, we will attempt to differentiate the thermal neutron capture and fast neutron scattered signals from gamma radiation signals through pulse shape discrimination using the PPO (and newer dopants) over-doping method.

## Methods

Both microwave and conventional syntheses are described in supporting information.

### Characterization

All reagents were purchased from either Sigma Aldrich, Frontier Scientific, or TCI America unless otherwise noted. ^1^H and ^13^C NMR spectra were obtained on a JEOL ECA 500 liquid-state NMR spectrometer and data obtained was manipulated in ACD/NMR processor software.

X-ray data were collected on a Bruker-Nonius X8 Proteum CCD diffractometer using Cu*Kα* radiation. The structures were solved using SHELXT and refined using SHELXL programs^39^. Molecular fragment editing, including the construction of suitable disorder models was performed using the XP program of SHELXTL. Hydrogen atoms were included using a riding model. The final models were checked using an R-tensor^38^, and by validation routines of the Platon program^40^ as implemented in the IUCr checkCIF facility.

Mass spectrometric measurements were acquired in positive-ion and negative-ion modes with a Bruker Ultraflextreme MALDI-TOF mass spectrometer (Bruker Daltonics, Billerica, MA) equipped with a 355 nm Nd:YAG laser. Spectra were collected in reflector mode with a grid voltage of 50.3%, and a low mass cutoff of 200 Da. Five replicate spectra were collected for each analysis as 100 shot composites at a sampling frequency of 1 kHz using automated laser rastering.

Molecular weight and molecular weight distributions of polymer samples were determined by gel permeation chromatography (GPC) using stabilized tetrahydrofuran (THF) as the eluent with a flow rate of 1.0 mL/min (Viscotek GPC pump; PLgel 5 um MIXED-C and MIXED-D columns: molecular weight range 200–2,000,000 and 200–400,000 g/mol (PS equiv), respectively.

Solid scintillator samples were tightly wrapped in white Teflon tape on all sides but one and attached to a Hamamatsu PMT (H2431-50) with silicone optical grease. The whole assembly was wrapped in aluminum foil and sealed with light-tight electrical tape. The PMT was biased using standard electronics and read out with a custom built waveform digitizer and DAQ system controlled by a MIDAS interface^44^. Samples were subjected to gamma radiation from a ^137^Cs source to quantify general scintillation response. A ^244^Cm/^13^C neutron-gamma source was tested in both a polyethylene cave to produce a high thermal neutron flux, as well as a lead cave, for fast neutron and gamma response.

### Preparation of samples

Azobisisobutyronitrile (AIBN) was recrystallized twice from methanol. The inhibitor in 4-vinyltoluene was removed by filtering through a 100 mg plug of a potassium carbonate and basic alumina mixture. An example of a typical plastic scintillator disc preparation is as follows. In a 120 mL clear glass bottle, the calculated amounts of 2,5-diphenyl oxazole (PPO), 1,4-bis(5-phenyloxazol-2-yl) benzene (POPOP), boron based materials, and AIBN were dissolved in the liquid 4-vinyltoluene monomer. The clear solution was degassed by gently bubbling with argon gas for 15–30 min. The solution was then bulk polymerized in an oil bath or an argon-filled vacuum oven at 80 °C for 96 hours, followed by 90 °C for 12 hours. The sample was cooled to room temperature and the glass bottle was broken with a mallet, giving a clear polymer disk (Figs 5, 7 and S15–16) of scintillating polymer. The sample was machined down to one flat side (meniscus side) using 100 grit sandpaper by hand or by belt depending on its mechanical and thermal stability. Then, the sample was polished using 150, 220, 300, 400, 600 and 600 wet-grit sandpapers. The final touches of polishing was done on a loose-cotton buffer wheel using white abrasive polishing compound and finished with blue buffing compound. Each sample has 4.7 cm diameter and 1.1–1.3 cm thickness.

## Additional Information

**How to cite this article**: Yemam, H. A. *et al.* Boron-rich benzene and pyrene derivatives for the detection of thermal neutrons. *Sci. Rep.* **5**, 13401; doi: 10.1038/srep13401 (2015).

## Supplementary Material

Supplementary Information

## Figures and Tables

**Table 1:** Comparison of multiple borylation by conventional heating vs microwave synthesis.

	**B_2_Pin_2_[equiv]**	**KOAc [equiv]**	**Yield [%]**	
			**Microwave**	**Conventional**
1	1.5	3.0	68	75
2	3.0	6.0	63	79
3	6.0	10.0	85	87
4	4.5	7.5	61	69
5	4.5	7.5	83	97
6	6.0	10.0	41	36

3–4 mol% Pd catalyst was used to synthesize 1–6.

**Table 2:** Plastic scintillator formulations.

**Sample**^a^	**Vinyl toluene[%wt]**	**Primary dopant**		**Wavelength shifter**		**Boron source**	
		**Name**	**[%wt]**	**Name**	**[%wt]**	**Name**	**[%wt]**
i	98.9	PPO	1.0	POPOP	0.1	—	—
ii	98.4	PPO	1.0	POPOP	0.1	135TrBB	0.5
iii	97.9	PPO	1.0	POPOP	0.1	135TrBB	1.0
iv	93.9	PPO	1.0	POPOP	0.1	135TrBB	5.0
v	97.9	PPO	1.0	POPOP	0.1	124TrBB	1.0
vi	93.9	PPO	1.0	POPOP	0.1	124TrBB	5.0
vii	97.9	PPO	1.0	POPOP	0.1	TBB	1.0
viii	93.9	PPO	1.0	POPOP	0.1	TBB	5.0
ix	98.9	TBP^b^	1.0	POPOP	0.1	TBP	1.0
x	97.9	TBP^b^	2.0	POPOP	0.1	TBP	2.0
xi	97.9	PPO	1.0	POPOP	0.1	TBP	1.0

^a^Total mass of each sample: 20.0 g.

^b^TBP acting as boron source and primary dopant.

**Table 3:** Light output, boron capture and polymer properties of plastic scintillator samples.

**Samples**	**Comparison to EJ 204**	**B content [%wt]**	^**10**^**B content [%w]**	**Neutron capture [keVee]**	**Mn [MDa]**	**Mw [MDa]**	**PDI**
i	95	—	—	—	1.37	3.52	2.57
ii	74	0.035	0.007	No capture	1.20	2.36	1.96
iii	78	0.070	0.014	78.8 ± 0.8	1.29	3.44	2.66
iv	78	0.356	0.071	73.1 ± 2.0	0.69	1.54	2.24
v	77	0.070	0.014	72.1 ± 2.6	0.91	2.49	2.73
vi	81	0.356	0.071	91.9 ± 0.2	1.34	2.88	2.22
vii	87	0.075	0.015	74.9 ± 2.0	0.32	0.81	2.58
viii	76	0.370	0.074	82.6 ± 0.7	1.26	4.39	3.47
ix	36	0.065	0.013	No capture	1.35	3.38	2.51
x	31	0.130	0.026	No capture	0.79	1.96	2.49
xi	70	0.065	0.013	69.8 ± 1.1	1.72	4.72	2.75
