# Supplementary material for: Boron-rich benzene and pyrene derivatives for the detection of thermal neutrons
Source: Sci Rep. 2015 Sep 3;5:13401. doi: 10.1038/srep13401 (PMC4558605; doi:10.1038/srep13401)
Supplement: Supplementary Information [file srep13401-s1.doc]

Supporting Information

**Boron-rich benzene and pyrene derivatives for the detection of thermal neutrons**

*Henok A Yemam,^1^ Adam Mahl,^2^ Unsal Koldemir,^1^ Tyler Remedes,^2^ Sean Parkin,^3^ Uwe Greife,^2^ Alan Sellinger ^1,*^*

1. Department of Chemistry and Geochemistry, Colorado School of Mines, Golden, CO 80401, USA
2. Department of Physics, Colorado School of Mines, Golden, CO 80401, USA
3. Department of Chemistry, University of Kentucky, Lexington, KY 40506, USA

*Corresponding author: [aselli@mines.edu](mailto:aselli@mines.edu)

Contents

1. Experimental Section S2-S7
2. NMR spectra S8-13
3. MALDI-TOF-MS S14
4. Absorption and Fluorescence spectra S14-15
5. Scintillation and Neutron Capture Efficiency S15-18
6. Sample Images S19

7. Crystallographic data for C_46_ H_68_ B_4_ O_8_ S20

8. Crystallographic Information File (CIF) for C_46_ H_68_ B_4_ O_8_ S21-43

**1. Experimental Section**

1. Experimental conditions (conventional heating)

**4,4,5,5-tetramethyl-2-(pyren-1-yl)-1,3,2-dioxaborolane (1)**

In a flame-dried Schlenk tube under argon atmosphere, 1-bromopyrene (200mg, 0.71mmol) and B_2_Pin_2_ (270mg, 1.07mmol) were dissolved in 20ml 1,4-dioxane. Flame dried KOAc (210 mg, 2.13mmol) was added quickly to the mixture followed by PdCl_2_(dppf) (17mg, 0.021mmol). The mixture was heated at 90°C overnight. Once the reaction was complete, the mixture was cooled to room temperature and 20-30ml ethyl acetate was added to quench the reaction. 30 ml DI water was added to extract the aqueous phase, 30 ml brine (2X) was used to wash the organic phase, and it was dried over anhydrous MgSO_4_ and filtered. After concentrating down the filtrate, silica column chromatography with hexanes as eluent gave a yellow powder (175mg, 75%). ^1^H NMR (300.0 MHz, CDCl_3_): δ 9.16 (d,1H; CH), δ 8.02-8.28 (m, 7H; CH), δ 1.54 (s, 12H; CH_3_). ^13^C {^1^H} NMR (75.5 MHz, CDCl_3_): δ 124.02-136.41 (CH), δ 83.82 (CCH_3_), δ 25.00 (CH_3_), n.o. (CB).

**1,6-bis(4,4,5,5-tetramethyl-1,3,2-dioxaborolan-2-yl)pyrene (2)**

In a flame-dried Schlenk tube under argon atmosphere, 1,6-dibrompyrene (200mg, 0.56mmol) and B_2_Pin_2_ (420mg, 1.67mmol) were dissolved in 20ml 1,4-dioxane. Flame dried KOAc (327mg, 3.32mmol) was added quickly to the mixture followed by PdCl_2_(dppf) (13.6mg, 0.017mmol). The mixture was heated at 90°C overnight. A scoop of catalyst (5mg) was added to complete the reaction. Once the reaction was complete, the mixture was cooled to room temperature and 20-30ml ethyl acetate was added to quench the reaction. 30 ml DI water was added to extract the aqueous phase, 30 ml brine (2X) was used to wash the organic phase, and it was dried over anhydrous MgSO_4_ and filtered. After concentrating down the filtrate, silica column chromatography with hexanes:dichloromethane (1:1) mixture as eluent gave a yellow powder (200mg, 79%). ^1^H NMR (300.0 MHz, CDCl_3_): δ 9.12 (d,2H; CH), δ 8.56 (d,2H; CH), δ 8.21(d,2H; CH), δ 8.15 (d,2H; CH), δ 1.51 (s, 24H; CH_3_). ^13^C {^1^H} NMR (75.5 MHz, CDCl_3_): δ 133.77 (CH), δ 133.20 (CH), δ 129.22 (CH), δ 127.99, δ 124.55 (CH), δ 83.96 (CCH_3_), δ 25.12 (CH_3_), n.o. (CB). MALDI TOF MS: m/z 455 (M^+^), 353, 326 (M-Bpin)^+^ .

**1,3,6,8-tetrakis(4,4,5,5-tetramethyl-1,3,2-dioxaborolan-2-yl)pyrene (3) (TBP)**

In a flame-dried Schlenk tube under argon atmosphere, 1,3,6,8-tetrabromopyrene (500mg, 0.97mmol) and B_2_Pin_2_ (1.47g, 5.79mmol) were dissolved in 20ml 1,4-dioxane. Flame dried KOAc (952mg, 9.7mmol) was added quickly to the mixture followed by PdCl_2_(dppf) (24mg, 0.029mmol). The mixture was heated at 90°C overnight. At the 16hr mark, 10mg catalyst was added to complete the reaction. Once complete, the mixture was cooled to room temperature and 20-30ml ethyl acetate was added to quench the reaction. 30 ml DI water was added to extract the aqueous phase, 30 ml brine (2X) was used to wash the organic phase, and it was dried over anhydrous MgSO_4_ and filtered. After concentrating down the filtrate, silica column chromatography with hexanes:ethyl acetate (8:2) mixture as eluent gave a yellow powder (592mg, 87%). MALDI TOF MS: m/z 707 (M^+^), 581 (M-Bpin)^+^, 454(M-2Bpin)^+^, 227 (M-3Bpin)^+^.

**1,3,5-tris(4,4,5,5-tetramethyl-1,3,2-dioxaborolan-2-yl)benzene (4) (135TrBB)**

In a flame-dried Schlenk tube under argon atmosphere, 1,3,5-tribromobenzene (200mg, 0.64mmol) and B_2_Pin_2_ (731.3mg, 2.88mmol) were dissolved in 20ml 1,4-dioxane. Flame dried KOAc (471.1mg, 4.8mmol) was added quickly to the mixture followed by PdCl_2_(dppf) (15.5mg, 0.029mmol). The mixture was heated at 90°C overnight. The progress of reaction was tracked by TLC (9 hexanes: 1 ethyl acetate) and visualization was achieved in an iodine chamber. Two spots (Rf 0.20 and Rf 0.25) were observed and heating was stopped and allowed to cool down to room temperature. 20-30ml ethyl acetate was added to quench the reaction. 30 ml DI water was added to extract the aqueous phase and 30 ml brine (2X) was used to wash the organic phase, dried over anhydrous MgSO_4_ and filtered. After concentrating down the filtrate, hexanes:ethyl acetate (9:1) mixture was used to elute Rf 0.25 in silica column chromatography to yield white powder [69%, 200mg]. ^1^H NMR (300.0 MHz, CDCl_3_): δ 8.36 (s,3H; CH), δ 1.32 (s, 36H; CH_3_). ^13^C {^1^H} NMR (75.5 MHz, CDCl_3_): δ 144.14 (CH), δ 83.79 (CCH_3_), δ 24.96 (CH_3_), n.o. (CB).

**2,2',2''-(benzene-1,2,4-triyl)tris(4,4,5,5-tetramethyl-1,3,2-dioxaborolane) (5) (124TrBB)**

In a flame-dried Schlenk tube under argon atmosphere, 1,2,4-tribromobenzene (200mg, 0.64mmol) and B_2_Pin_2_ (731.3mg, 2.88mmol) were dissolved in 20ml 1,4-dioxane. Flame dried KOAc (471.1mg, 4.8mmol) was added quickly to the mixture followed by PdCl_2_(dppf) (15.5mg, 0.029mmol). The mixture was heated at 90°C overnight. The progress of reaction was tracked by TLC (9 hexanes: 1 ethyl acetate) and visualization was achieved in an iodine chamber. Two spots (Rf 0.20 and Rf 0.25) were observed and heating was stopped to allow cooling to room temperature. 20-30ml ethyl acetate was added to quench the reaction. 30 ml DI water was added to extract the aqueous phase, 30 ml brine (2X) was used to wash the organic phase, and it was dried over anhydrous MgSO_4_ and filtered. After concentrating down the filtrate, hexanes:ethyl acetate (9:1) mixture was used to elute Rf 0.2 by silica column chromatography to yield a white powder (280mg, 96%). ^1^H NMR at 80^o^C (300.0 MHz, DMSO-d): δ 7.91 (s,1H; CH), δ 7.70 (d,1H; CH) , δ 7.52 (d,1H; CH), δ 1.34 (s, 12H; CH_3_), δ 1.33 (s, 12H; CH_3_), δ 1.19 (s, 12H; CH_3_). ^13^C {^1^H} NMR (75.5 MHz, CDCl_3_): δ 139.48 (CH), δ 135.47 (CH), δ 132.41 (CH), δ 83.44 (CCH_3_), δ 83.69 (CCH_3_), δ 83.480 (CCH_3_), δ 25.02 (CH_3_), n.o. (CB).

**1,2,4,5-tetrakis(4,4,5,5-tetramethyl-1,3,2-dioxaborolan-2-yl)benzene (6) (TBB)**

In a flame-dried Schlenk tube under argon atmosphere, 1,2,4,5-tetrabromobenzene (380mg, 0.97mmol) and B_2_Pin_2_ (1.48g, 5.82mmol) were dissolved in 20ml 1,4-dioxane. Flame dried KOAc (952.1mg, 9.70mmol) was added quickly to the mixture followed by PdCl_2_(dppf) (23.6mg, 0.030mmol). The mixture was heated at 90^o^C overnight. The progress of reaction was tracked by TLC (9 hexanes: 1 ethyl acetate) and visualization was achieved in an iodine chamber. Two spots (Rf 0.20 and Rf 0.25) were observed and heating was stopped to allow cooling to room temperature. 20-30ml ethyl acetate was added to quench the reaction. 30 ml DI water was added to extract the aqueous phase, 30 ml brine (2X) was used to wash the organic phase, and it was dried over anhydrous MgSO_4_ and filtered. After concentrating down the filtrate, hexanes:ethyl acetate (9:1) mixture was used to elute Rf 0.25 in silica column chromatography to yield a white powder. Alternatively, after concentration of the filtrate, both fractions were collected by filtering through a silica chromatography column, with elution by hexanes: ethyl acetate (7: 3) mixture. The mixture was vacuum dried and 20 ml hexanes was added and left in the fridge overnight. The precipitate was collected by filtration (202mg, 36%). ^1^H NMR (300.0 MHz, CDCl_3_): δ 7.89 (s,2H; CH), δ 1.36 (s, 36H; CH_3_). ^13^C {^1^H} NMR (75.5 MHz, CDCl_3_): δ 137.90 (CH), δ 83.84 (CCH_3_), δ 24.99 (CH_3_), n.o. (CB).

2. Experimental conditions (microwave synthesis)

**4,4,5,5-tetramethyl-2-(pyren-1-yl)-1,3,2-dioxaborolane (1)**

1-bromopyrene (500mg, 1.78mmol) and B_2_Pin_2_ (678.0mg, 2.67mmol) were added to a 20ml microwave flask. Flame dried KOAc (524mg, 5.33mmol) and PdCl_2_(dppf) (58mg, 0.071mmol) were added quickly to the flask. After adding 12ml 1,4-dioxane, the flask was capped tightly and run at 130°C for 40min. Once the reaction was complete, the mixture was cooled to room temperature and 15ml ethyl acetate was added to quench the reaction. 20 ml DI water was added to extract the aqueous phase and 20 ml brine (2X) was used to wash the organic phase. It was dried over anhydrous MgSO_4_ and filtered. After concentrating down the filtrate, silica column chromatography with hexanes as eluent gave a yellow powder (396 mg, 68%).

**1,6-bis(4,4,5,5-tetramethyl-1,3,2-dioxaborolan-2-yl)pyrene (2)**

1,6-dibromopyrene (500mg, 1.39mmol) and B_2_Pin_2_ (1.06g, 4.17mmol) were added to a 20ml microwave flask. Flame dried KOAc (818mg, 8.33mmol) and PdCl_2_(dppf) (45.4mg, 0.056mmol) were added quickly to the flask. After adding 12ml 1,4-dioxane, the flask was capped tightly and run at 130°C for 40min. Once the reaction was complete, the mixture was cooled to room temperature and 15ml ethyl acetate was added to quench the reaction. 20 ml DI water was added to extract the aqueous phase and 20 ml brine (2X) was used to wash the organic phase. It was dried over anhydrous MgSO_4_ and filtered. After concentrating down the filtrate, silica column chromatography with a mixture of hexanes and dichloromethane (1:1) as eluent yielded a yellow powder (400mg, 63%).

**1,3,6,8-tetrakis(4,4,5,5-tetramethyl-1,3,2-dioxaborolan-2-yl)pyrene (3) (TBP)**

1,3,6,8-tetrabromopyrene (500mg, 0.97mmol) and B_2_Pin_2_ (1.48g, 5.82mmol) were added to a 20ml microwave flask. Flame dried KOAc (952.0mg, 9.70mmol) and PdCl_2_(dppf) (31.5mg, 0.039mmol) were added quickly to the flask. After adding 12ml 1,4-dioxane, the flask was capped tightly and run at 130°C for 1hr. Once the reaction was complete, the mixture was cooled to room temperature and 15ml ethyl acetate was added to quench the reaction. 20 ml DI water was added to extract the aqueous phase and 20 ml brine (2X) was used to wash the organic phase. It was dried over anhydrous MgSO_4_ and filtered. After concentrating down the filtrate, silica column chromatography with a mixture of hexanes and ethyl acetate (8:2) as eluent yielded a yellow powder (579mg, 85%).

**1,3,5-tris(4,4,5,5-tetramethyl-1,3,2-dioxaborolan-2-yl)benzene(4) (135TrBB)**

1,3,5-tribromobenzene (500mg, 1.59mmol) and B_2_Pin_2_ (1.82g, 7.15mmol) were added to a 20ml microwave flask. Flame dried KOAc (1.17g, 11.9mmol) and PdCl_2_(dppf) (52mg, 0.064mmol) were added quickly to the flask. After adding 12ml 1,4-dioxane, the flask was capped tightly and run at 130°C for 1hr. TLC (9 hexanes: 1 ethyl acetate) showed there were two spots Rf 0.2 and Rf 0.25. The mixture was cooled to room temperature and 30ml ethyl acetate was added to quench the reaction. 30 ml DI water was added to extract the aqueous phase, 30 ml brine (2X) was used to wash the organic phase, and it was dried over anhydrous MgSO_4_ and filtered. After concentrating down the filtrate, hexanes:ethyl acetate (9:1) mixture was used to elute Rf 0.25 by silica column chromatography to yield a white powder (440mg, 61%).

**2,2',2''-(benzene-1,2,4-triyl)tris(4,4,5,5-tetramethyl-1,3,2-dioxaborolane) (5) (124TrBB)**

1,3,5-tribromobenzene (500mg, 1.59mmol) and B_2_Pin_2_ (1.82g, 7.15mmol) were added to a 20ml microwave flask. Flame dried KOAc (1.17g, 11.9mmol) and PdCl_2_(dppf) (51.9mg, 0.064mmol) were added quickly to the flask. After adding 12ml 1,4-dioxane, the flask was capped tightly and run at 130°C for 1hr. TLC (9 hexanes: 1 ethyl acetate) showed there were two spots Rf 0.2 and Rf 0.25. The mixture was cooled to room temperature and 30ml ethyl acetate was added to quench the reaction. 30 ml DI water was added to extract the aqueous phase and 30 ml brine (2X) was used to wash the organic phase, dried over anhydrous MgSO4 and filtered. After concentrating down the filtrate, hexanes:ethyl acetate (9:1) mixture was used to elute Rf 0.2 in silica column chromatography to yield a white powder (600mg, 83%).

**1,2,4,5-tetrakis(4,4,5,5-tetramethyl-1,3,2-dioxaborolan-2-yl)benzene (6) (TBB)**

1,2,4,5-tetrabromobenzene (500mg, 1.27mmol) and B_2_Pin_2_ (1.94g, 7.63mmol) were added to a 20ml microwave flask. Flame dried KOAc (1.25g, 12.7mmol) and PdCl_2_(dppf) (41.6mg, 0.051mmol) were added quickly to the flask. After adding 12ml 1,4-dioxane, the flask was capped tightly and run at 130°C for 1hr. TLC (9 hexanes: 1 ethyl acetate) showed there were two spots Rf 0.2 and Rf 0.25. The mixture was cooled to room temperature and 30ml ethyl acetate was added to quench the reaction. 30 ml DI water was added to extract the aqueous phase, 30 ml brine (2X) was used to wash the organic phase, and it was dried over anhydrous MgSO_4_ and filtered. After concentrating down the filtrate, hexanes:ethyl acetate (9:1) mixture was used to elute Rf 0.25 by silica column chromatography to yield a white powder. Alternatively, after filtrate concentration, both fractions were collected by filtering through a silica chromatography column, with elution by a hexanes: ethyl acetate (7: 3) mixture. The mixture was vacuum dried, and 20 ml hexanes was added and left in the fridge overnight. The precipitate was collected by filtration to yield a white powder (250mg, 41%).

**2. ^1^H and ^13^C NMR Spectra**

**Figure S1**. ^1^H and ^13^C NMR for monoborylatedpyrene.

**Figure S2.** ^1^H and ^13^C NMR for 1,6-diborylatedpyrene.

**Figure S3.** ^1^H and ^13^C NMR for 135TrBB.

**Figure S4**. ^1^H and ^13^C NMR for 124TrBB.

**Figure S5**. ^1^H and ^13^C NMR for TBB.

1. **MALDI-TOF MS**


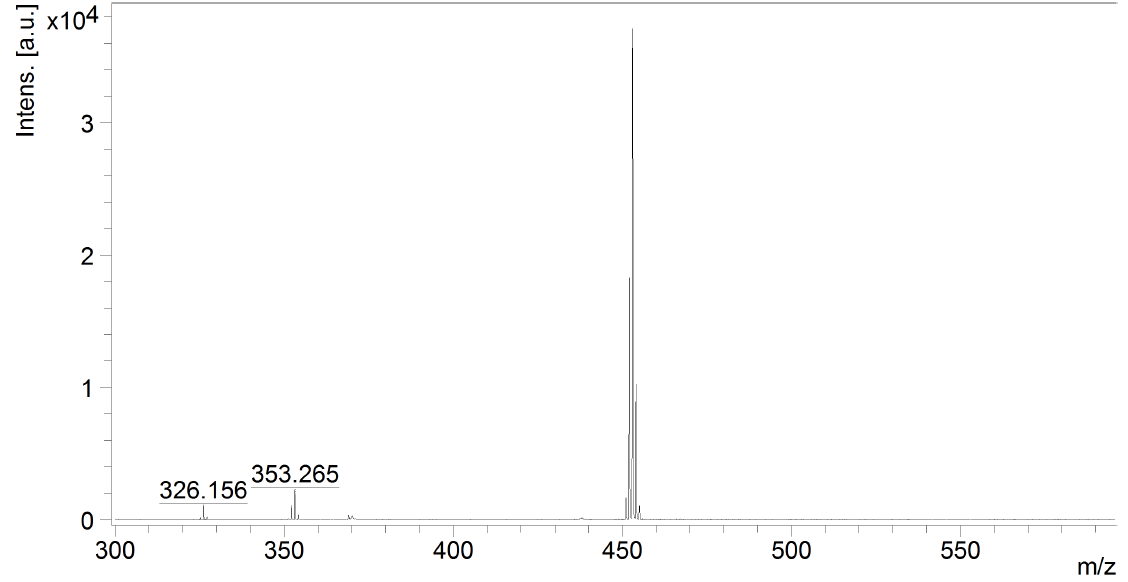


[M-Bpin]^+^

[M]^+^

**Figure S6**. MALDI-TOF-MS spectra for 1,6-diborylatedpyrene.

1. **Absorption and Emission spectra**

Absorption spectrometry was performed using a Varian spectrophotometer. Steady-state fluorescence measurements were conducted using HORIBA Jobin Yvon fluorolog, FL-1057. Stock solutions of TBP were made in ACS grade n-hexanes.


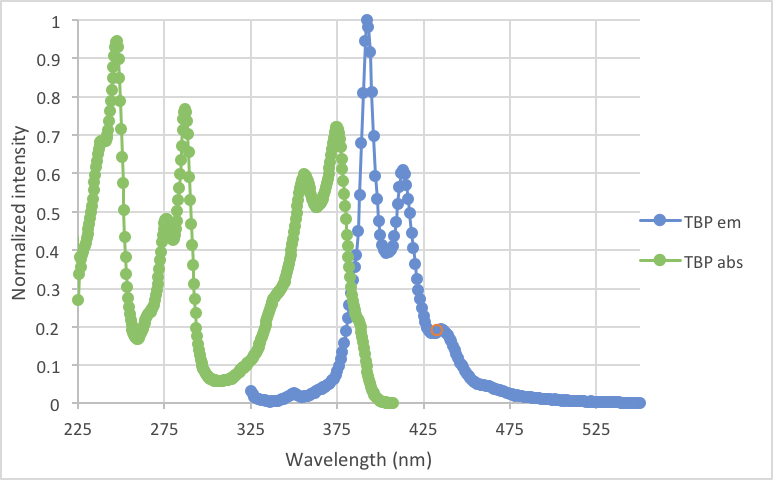


**Figure S7**. Optical absorption and emission measurement of TBP.


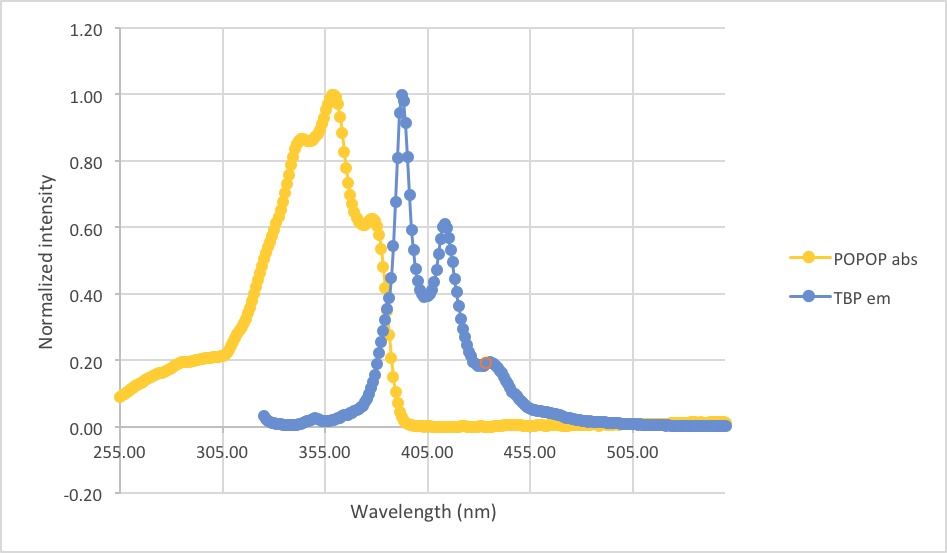


**Figure S8**. Unoptimized optical spectral overlap between emission of TBP and absorption of POPOP.

**5. Scintillation and Neutron Capture Efficiency**


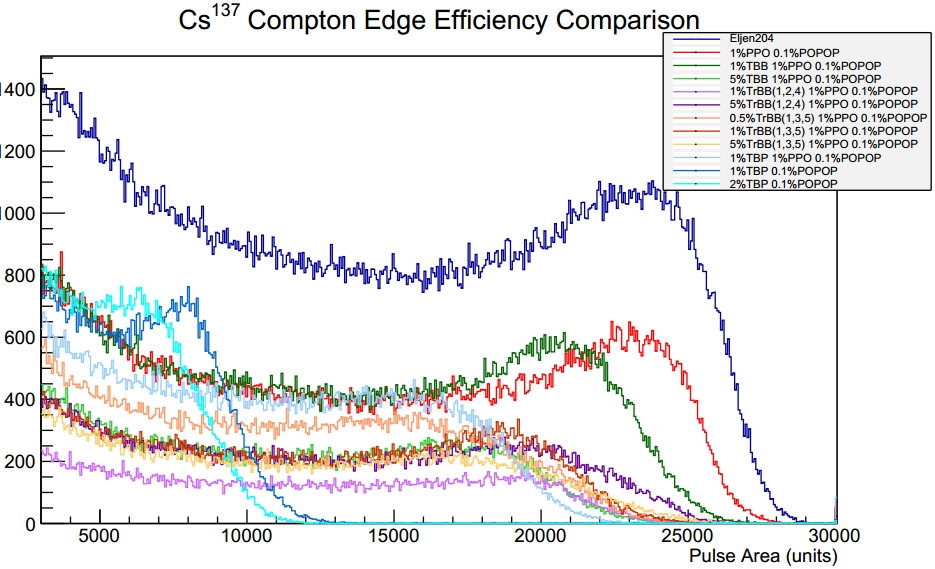


**Figure S9.** Light output comparison of plastic scintillator (i-xi) vs EJ-204.


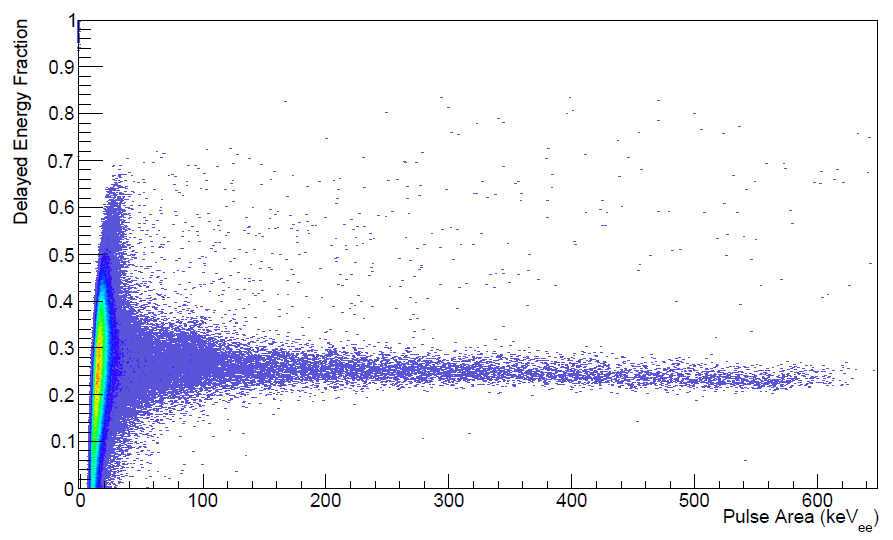


Figure S9: Response of plastic scintillator (1% 135TrBB) to gamma and neutron

**Figure S10**. Response of plastic scintillator (1% 135TrBB) to gamma and neutron source.


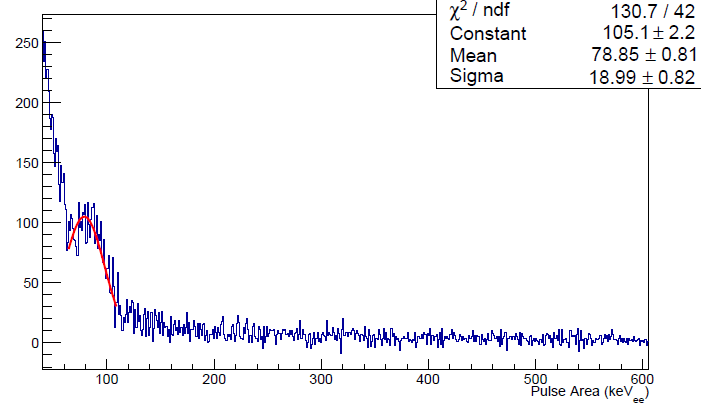


**Figure S11**. Response of plastic scintillator (1% 135 TrBB) with gamma shielding lead.


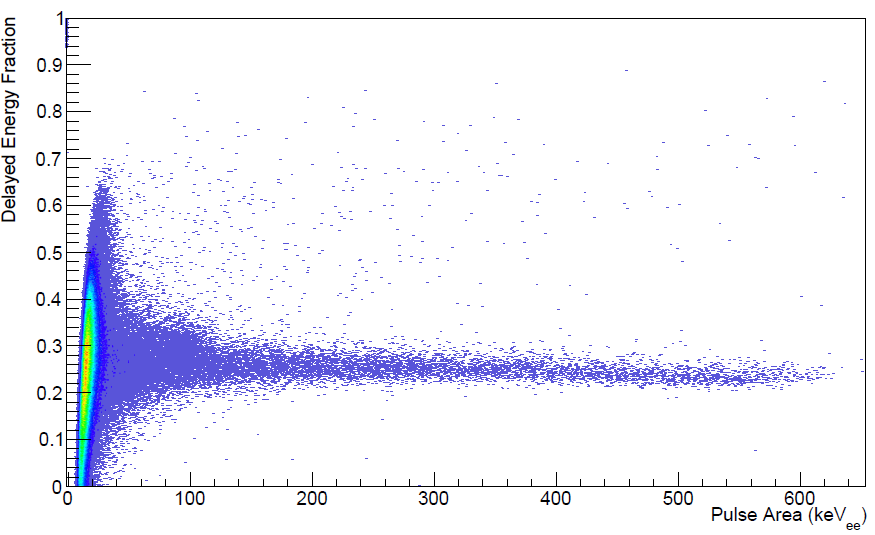


**Figure S12.** Response of plastic scintillator (5% 135TrBB) to gamma and neutron source.


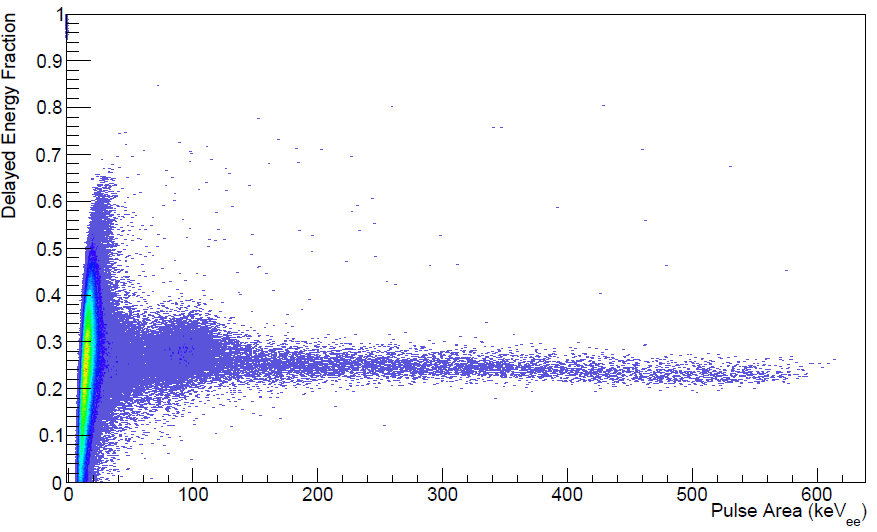


**Figure S13**. Response of plastic scintillator (5% 124TrBB) to gamma and neutron source.


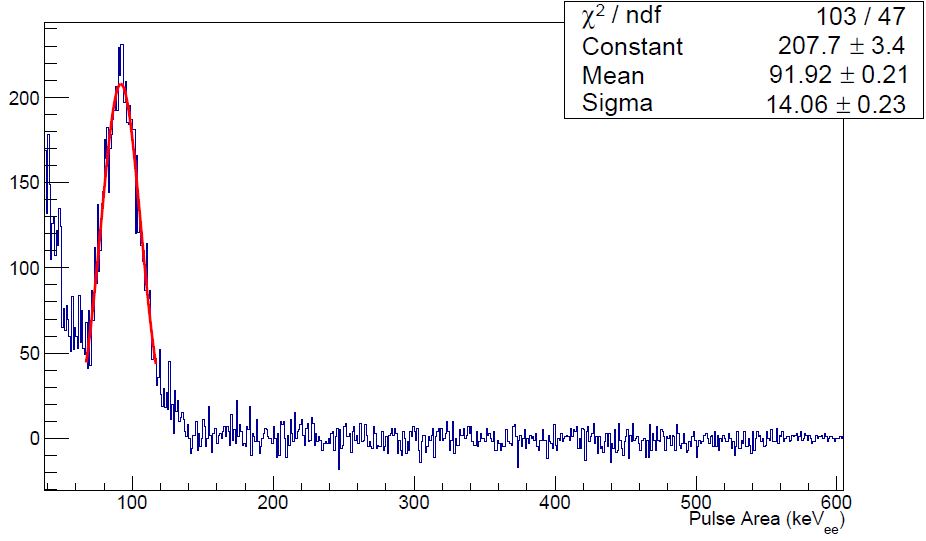


**Figure S14**. Response of plastic scintillator (5% 124 TrBB) with gamma shielding lead.

**6. Sample Images**


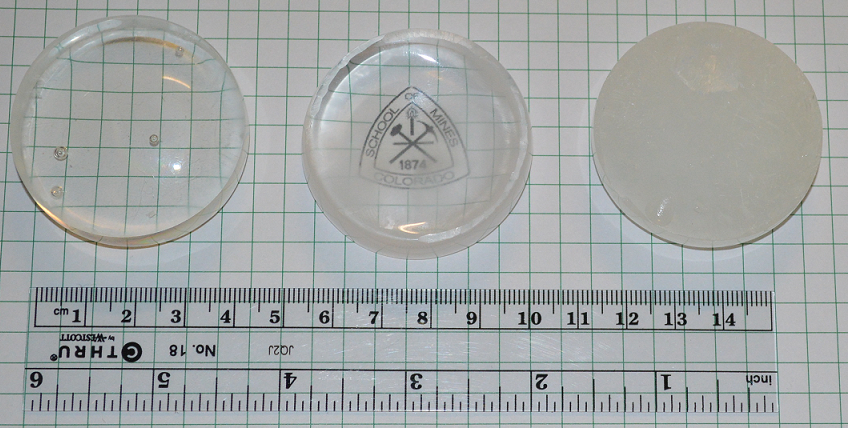


**Figure S15**. Left to right: Sample ii, iii and iv incorporating 0.5, 1.0 and 5% 135TrBB respectively.


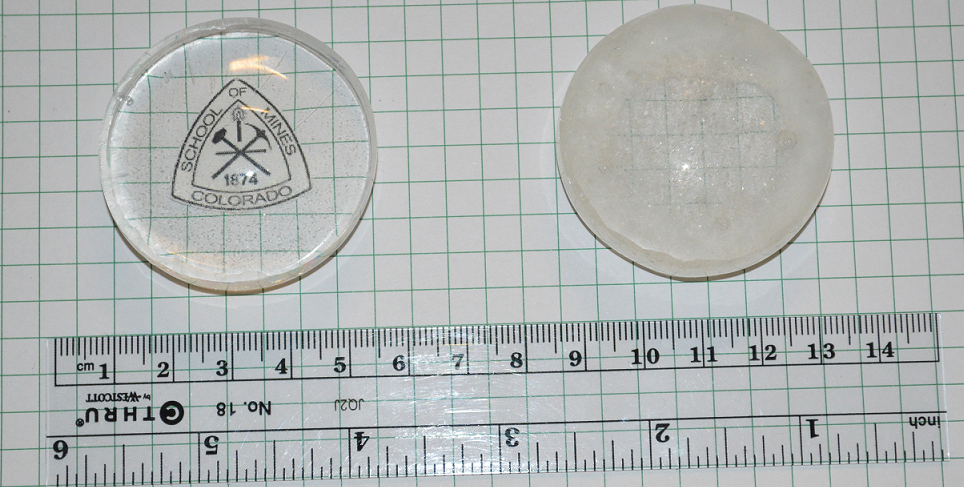


**Figure S16**. Left to right: Sample vii and viii incorporating 1 and 5% TBB respectively.

**7. Crystallographic data for C_46_ H_68_ B_4_ O_8_**

Formula weight 792.24

Temperature 210(2) K

Wavelength 1.54178 Å

Crystal system, space group Monoclinic, *I*2

Unit cell dimensions a = 16.4584(4) Å alpha = 90°.

b = 7.3229(2) Å beta = 96.2680(11)°.

c = 18.8669(7) Å gamma = 90°.

Volume 2260.31(12) A^3^

Z, Calculated density 2, 1.164 Mg/m^3^

Absorption coefficient 0.600 mm^-1^

F(000) 856

Crystal size 0.25 x 0.20 x 0.02 mm

Theta range for data collection 3.39 to 68.24°.

Limiting indices -19<=h<=14, -8<=k<=8, -22<=l<=21

Reflections collected / unique 15591 / 3702 [R(int) = 0.0431]

Completeness to theta = 67.68° 99.7 %

Absorption correction Semi-empirical from equivalents

Max. and min. transmission 0.984 and 0.873

Refinement method Full-matrix least-squares on F^2^

Data / restraints / parameters 3702 / 349 / 316

Goodness-of-fit on F^2 1.059

Final R indices [I>2sigma(I)] R_1_ = 0.0637, wR_2_ = 0.1997

R indices (all data) R_1_ = 0.0709, wR_2_ = 0.2093

Absolute structure parameter 0.5

Largest diff. peak and hole 0.348 and -0.491 e.Å^-3^

**8) Crystallographic Information File (CIF) for C_46_ H_68_ B_4_ O_8_**

data_x14361x

_audit_creation_method SHELXL-2014

# CIF file edited on-the-fly by script CIFFIX (S. Parkin, 2000-2014).

# CIFFIX: http://xray.uky.edu/people/parkin/programs/ciffix

_publ_section_exptl_refinement

;

H atoms were found in difference Fourier maps, but subsequently included

in the refinement using riding models, with constrained distances set to

0.94 %A (C~sp2~H), 0.97 %A (RCH~3~) and 0.98 %A (R~2~CH~2~).

U~iso~(H) parameters were set to values of either 1.2U~eq~ or 1.5U~eq~

(RCH~3~ only) of the attached atom.

To ensure satisfactory refinement of disordered groups in the structure,

a combination of constraints and restraints were employed. The constraints

(SHELXL commands EXYZ and EADP) were used to fix overlapping fragments.

Restraints were used to maintain the integrity of ill-defined or disordered

groups (SHELXL commands SAME, RIGU and ISOR).

The structure was refined as twinned across a mirror plane perpendicular to

the b-axis. This is entirely equivalent (in this case) to being twinned by

inversion (owing the the 2-fold). Twin component fractions were fixed at

50:50.

;

_publ_section_references

;

Bruker-AXS (2006).

<i>APEX2</i> Bruker-AXS Inc., Madison, Wisconsin, USA.

Hope, H. (1994).

<i>Prog. Inorg. Chem.</i> <b>41</b>, 1--19.

Parkin, S. & Hope, H. (1998).

<i>J. Appl. Cryst.</i> <b>31</b>, 945--953.

Parkin, S. (2000).

<i>Acta Cryst.</i> A<b>56</b>, 157--162.

Parkin, S. (2013).

<i>CIFFIX</i>, http://xray.uky.edu/people/parkin/programs/ciffix

Sheldrick, G.M. (2008a).

<i>Acta Cryst.</i> A<b>64</b>, 112--122.

Sheldrick, G.M. (2008b).

<i>SADABS</i> University of G\"ottingen, Germany.

Spek, A.L. (2009).

<i>Acta Cryst.</i> D<b>65</b>, 148--155.

;

_chemical_name_systematic

;

?

;

_chemical_name_common ?

_chemical_melting_point ?

_chemical_formula_moiety 'C40 H54 B4 O8, C6 H14'

_chemical_formula_sum 'C46 H68 B4 O8'

_chemical_formula_weight 792.24

loop_

_atom_type_symbol

_atom_type_description

_atom_type_scat_dispersion_real

_atom_type_scat_dispersion_imag

_atom_type_scat_source

'C' 'C' 0.0181 0.0091

'International Tables Vol C Tables 4.2.6.8 and 6.1.1.4'

'H' 'H' 0.0000 0.0000

'International Tables Vol C Tables 4.2.6.8 and 6.1.1.4'

'B' 'B' 0.0090 0.0039

'International Tables Vol C Tables 4.2.6.8 and 6.1.1.4'

'O' 'O' 0.0492 0.0322

'International Tables Vol C Tables 4.2.6.8 and 6.1.1.4'

_space_group_crystal_system monoclinic

_space_group_IT_number 5

_space_group_name_H-M_alt 'I 2'

_space_group_name_Hall 'I 2y'

_shelx_space_group_comment

;

The symmetry employed for this shelxl refinement is uniquely defined

by the following loop, which should always be used as a source of

symmetry information in preference to the above space-group names.

They are only intended as comments.

;

loop_

_space_group_symop_operation_xyz

'x, y, z'

'-x, y, -z'

'x+1/2, y+1/2, z+1/2'

'-x+1/2, y+1/2, -z+1/2'

_cell_length_a 16.4584(4)

_cell_length_b 7.3229(2)

_cell_length_c 18.8669(7)

_cell_angle_alpha 90

_cell_angle_beta 96.2680(11)

_cell_angle_gamma 90

_cell_volume 2260.31(12)

_cell_formula_units_Z 2

_cell_measurement_temperature 210(2)

_cell_measurement_reflns_used 7497

_cell_measurement_theta_min 3.39

_cell_measurement_theta_max 68.23

_exptl_crystal_description shard

_exptl_crystal_colour colourless

_exptl_crystal_density_meas ?

_exptl_crystal_density_method ?

_exptl_crystal_density_diffrn 1.164

_exptl_crystal_F_000 856

_exptl_transmission_factor_min ?

_exptl_transmission_factor_max ?

_exptl_crystal_size_max 0.250

_exptl_crystal_size_mid 0.200

_exptl_crystal_size_min 0.020

_exptl_absorpt_coefficient_mu 0.600

_shelx_estimated_absorpt_T_min 0.865

_shelx_estimated_absorpt_T_max 0.988

_exptl_absorpt_correction_type multi-scan

_exptl_absorpt_correction_T_min 0.873

_exptl_absorpt_correction_T_max 0.984

_exptl_absorpt_process_details '<i>SADABS</i> (Sheldrick, 2008b)'

_exptl_special_details

;

The crystal was mounted with polyisobutene oil on the tip of a fine glass

fibre, which was fastened in a copper mounting pin with electrical solder.

It was placed directly into the cold gas stream of a liquid nitrogen based

cryostat, according to published methods (Hope, 1994; Parkin & Hope, 1998).

The crystals underwent a non-destructive phase transition when cooled to

90K, resulting in many reflections being split in to three. These split

reflections eventually coalesce as the temperature rises, and were single

by about 200K. On further warming to around 230K, the diffraction pattern

seemed to get a little fuzzier. For this reason data were collected with

the crystal at 210K.

;

_diffrn_ambient_temperature 210(2)

_diffrn_radiation_wavelength 1.54178

_diffrn_radiation_type CuK\a

_diffrn_source 'fine-focus rotating anode'

_diffrn_measurement_device_type 'Bruker X8 Proteum diffractometer'

_diffrn_measurement_method '\f and \w scans'

_diffrn_detector_area_resol_mean 5.6

_diffrn_reflns_number 15591

_diffrn_reflns_av_unetI/netI 0.0352

_diffrn_reflns_av_R_equivalents 0.0431

_diffrn_reflns_limit_h_min -19

_diffrn_reflns_limit_h_max 14

_diffrn_reflns_limit_k_min -8

_diffrn_reflns_limit_k_max 8

_diffrn_reflns_limit_l_min -22

_diffrn_reflns_limit_l_max 21

_diffrn_reflns_theta_min 3.385

_diffrn_reflns_theta_max 68.238

_diffrn_reflns_theta_full 67.679

_diffrn_measured_fraction_theta_max 0.996

_diffrn_measured_fraction_theta_full 0.997

_diffrn_reflns_Laue_measured_fraction_max 0.996

_diffrn_reflns_Laue_measured_fraction_full 0.997

_diffrn_reflns_point_group_measured_fraction_max 0.997

_diffrn_reflns_point_group_measured_fraction_full 0.998

_reflns_number_total 3702

_reflns_number_gt 3212

_reflns_threshold_expression 'I > 2\s(I)'

_reflns_Friedel_coverage 0.847

_reflns_Friedel_fraction_max 0.999

_reflns_Friedel_fraction_full 1.000

_reflns_special_details

;

Reflections were merged by SHELXL according to the crystal

class for the calculation of statistics and refinement.

_reflns_Friedel_fraction is defined as the number of unique

Friedel pairs measured divided by the number that would be

possible theoretically, ignoring centric projections and

systematic absences.

;

_computing_data_collection '<i>APEX2</i> (Bruker-AXS, 2006)'

_computing_cell_refinement '<i>APEX2</i> (Bruker-AXS, 2006)'

_computing_data_reduction '<i>APEX2</i> (Bruker-AXS, 2006)'

_computing_structure_solution '<i>SHELXS-97</i> (Sheldrick, 2008a)'

_computing_structure_refinement '<i>SHELXL-2014</i> (Sheldrick, 2008a)'

_computing_molecular_graphics '<i>XP in SHELXTL</i> (Sheldrick, 2008a)'

_computing_publication_material

'<i>SHELX</i> (Sheldrick, 2008a) and <i>CIFFIX</i> (Parkin, 2013)'

_refine_special_details

;

Refinement progress was checked using <i>Platon</i> (Spek, 2009) and by

an <i>R</i>-tensor (Parkin, 2000). The final model was further checked

with the IUCr utility <i>checkCIF</i>.

;

_refine_ls_structure_factor_coef Fsqd

_refine_ls_matrix_type full

_refine_ls_weighting_scheme calc

_refine_ls_weighting_details

'w=1/[\s^2^(Fo^2^)+(0.1298P)^2^+1.6782P] where P=(Fo^2^+2Fc^2^)/3'

_atom_sites_solution_primary direct

_atom_sites_solution_secondary difmap

_atom_sites_solution_hydrogens difmap

_refine_ls_hydrogen_treatment constr

_refine_ls_extinction_method none

_refine_ls_extinction_coef .

_refine_ls_abs_structure_details

;

Refined as twinned across a mirror plane perpendicular to the b-axis.

;

# The molecule is achiral, which means that 'absolute configuration' has

# no relevance for this structure.

_refine_ls_abs_structure_Flack .

_chemical_absolute_configuration .

_refine_ls_number_reflns 3702

_refine_ls_number_parameters 316

_refine_ls_number_restraints 349

_refine_ls_R_factor_all 0.0709

_refine_ls_R_factor_gt 0.0637

_refine_ls_wR_factor_ref 0.2093

_refine_ls_wR_factor_gt 0.1997

_refine_ls_goodness_of_fit_ref 1.059

_refine_ls_restrained_S_all 1.037

_refine_ls_shift/su_max 0.001

_refine_ls_shift/su_mean 0.000

loop_

_atom_site_label

_atom_site_type_symbol

_atom_site_fract_x

_atom_site_fract_y

_atom_site_fract_z

_atom_site_U_iso_or_equiv

_atom_site_adp_type

_atom_site_occupancy

_atom_site_site_symmetry_order

_atom_site_calc_flag

_atom_site_refinement_flags_posn

_atom_site_refinement_flags_adp

_atom_site_refinement_flags_occupancy

_atom_site_disorder_assembly

_atom_site_disorder_group

C1 C 0.43233(16) 0.5079(10) 0.67000(13) 0.0298(6) Uani 1 1 d . . . . .

H1 H 0.4142 0.5032 0.7155 0.036 Uiso 1 1 calc R U . . .

C2 C 0.37360(15) 0.5118(10) 0.61020(13) 0.0280(6) Uani 1 1 d . . . . .

C3 C 0.40072(15) 0.5114(10) 0.54187(13) 0.0260(6) Uani 1 1 d . . . . .

C4 C 0.48610(14) 0.5115(10) 0.53475(12) 0.0242(6) Uani 1 1 d . . . . .

C5 C 0.54378(15) 0.5103(10) 0.59695(13) 0.0257(6) Uani 1 1 d . . . . .

C6 C 0.51604(16) 0.5106(10) 0.66516(13) 0.0292(6) Uani 1 1 d . . . . .

C7 C 0.34493(15) 0.5103(10) 0.47767(13) 0.0300(6) Uani 1 1 d . . . . .

H7 H 0.2884 0.5103 0.4813 0.036 Uiso 1 1 calc R U . . .

C8 C 0.37099(15) 0.5092(10) 0.41264(13) 0.0305(6) Uani 1 1 d . . . . .

H8 H 0.3322 0.5077 0.3722 0.037 Uiso 1 1 calc R U . . .

B1 B 0.28178(19) 0.5112(11) 0.62453(16) 0.0349(7) Uani 0.528(6) 1 d D U P A 1

O1 O 0.2585(3) 0.5554(12) 0.6896(3) 0.0404(13) Uani 0.528(6) 1 d D U P A 1

O2 O 0.2166(3) 0.4562(11) 0.5764(3) 0.0385(8) Uani 0.528(6) 1 d D U P A 1

C9 C 0.1686(4) 0.5663(9) 0.6808(4) 0.0434(13) Uani 0.528(6) 1 d D U P A 1

C10 C 0.1462(3) 0.4398(9) 0.6163(4) 0.0427(11) Uani 0.528(6) 1 d D U P A 1

C11 C 0.1366(8) 0.518(9) 0.7493(5) 0.061(2) Uani 0.528(6) 1 d D U P A 1

H11A H 0.0774 0.5252 0.7434 0.092 Uiso 0.528(6) 1 calc R U P A 1

H11B H 0.1533 0.3946 0.7628 0.092 Uiso 0.528(6) 1 calc R U P A 1

H11C H 0.1583 0.6024 0.7862 0.092 Uiso 0.528(6) 1 calc R U P A 1

C12 C 0.1481(11) 0.7673(19) 0.6628(8) 0.061(3) Uani 0.528(6) 1 d D U P A 1

H12A H 0.0892 0.7830 0.6564 0.092 Uiso 0.528(6) 1 calc R U P A 1

H12B H 0.1714 0.8447 0.7015 0.092 Uiso 0.528(6) 1 calc R U P A 1

H12C H 0.1706 0.8007 0.6192 0.092 Uiso 0.528(6) 1 calc R U P A 1

C13 C 0.1426(11) 0.2396(19) 0.6383(7) 0.058(3) Uani 0.528(6) 1 d D U P A 1

H13A H 0.0964 0.2210 0.6653 0.087 Uiso 0.528(6) 1 calc R U P A 1

H13B H 0.1364 0.1633 0.5960 0.087 Uiso 0.528(6) 1 calc R U P A 1

H13C H 0.1927 0.2072 0.6676 0.087 Uiso 0.528(6) 1 calc R U P A 1

C14 C 0.0712(5) 0.488(4) 0.5679(7) 0.057(4) Uani 0.528(6) 1 d D U P A 1

H14A H 0.0236 0.4774 0.5937 0.086 Uiso 0.528(6) 1 calc R U P A 1

H14B H 0.0757 0.6119 0.5509 0.086 Uiso 0.528(6) 1 calc R U P A 1

H14C H 0.0656 0.4046 0.5276 0.086 Uiso 0.528(6) 1 calc R U P A 1

B1' B 0.28178(19) 0.5112(11) 0.62453(16) 0.0349(7) Uani 0.472(6) 1 d D U P A 2

O1' O 0.2579(4) 0.4685(14) 0.6895(3) 0.0404(13) Uani 0.472(6) 1 d D U P A 2

O2' O 0.2176(3) 0.5760(12) 0.5776(3) 0.0385(8) Uani 0.472(6) 1 d D U P A 2

C9' C 0.1681(4) 0.4568(9) 0.6800(4) 0.0434(13) Uani 0.472(6) 1 d D U P A 2

C10' C 0.1456(3) 0.5836(9) 0.6157(4) 0.0427(11) Uani 0.472(6) 1 d D U P A 2

C11' C 0.1352(9) 0.504(10) 0.7481(6) 0.061(2) Uani 0.472(6) 1 d D U P A 2

H11D H 0.0760 0.4964 0.7417 0.092 Uiso 0.472(6) 1 calc R U P A 2

H11E H 0.1566 0.4195 0.7850 0.092 Uiso 0.472(6) 1 calc R U P A 2

H11F H 0.1515 0.6275 0.7619 0.092 Uiso 0.472(6) 1 calc R U P A 2

C12' C 0.1491(13) 0.255(2) 0.6615(8) 0.058(3) Uani 0.472(6) 1 d D U P A 2

H12D H 0.0904 0.2378 0.6544 0.087 Uiso 0.472(6) 1 calc R U P A 2

H12E H 0.1725 0.2233 0.6181 0.087 Uiso 0.472(6) 1 calc R U P A 2

H12F H 0.1725 0.1782 0.7002 0.087 Uiso 0.472(6) 1 calc R U P A 2

C13' C 0.1365(12) 0.783(2) 0.6376(8) 0.061(3) Uani 0.472(6) 1 d D U P A 2

H13D H 0.0889 0.7951 0.6634 0.092 Uiso 0.472(6) 1 calc R U P A 2

H13E H 0.1849 0.8205 0.6680 0.092 Uiso 0.472(6) 1 calc R U P A 2

H13F H 0.1298 0.8591 0.5953 0.092 Uiso 0.472(6) 1 calc R U P A 2

C14' C 0.0729(6) 0.529(5) 0.5654(7) 0.057(4) Uani 0.472(6) 1 d D U P A 2

H14D H 0.0242 0.5336 0.5900 0.086 Uiso 0.472(6) 1 calc R U P A 2

H14E H 0.0669 0.6127 0.5253 0.086 Uiso 0.472(6) 1 calc R U P A 2

H14F H 0.0806 0.4061 0.5484 0.086 Uiso 0.472(6) 1 calc R U P A 2

B2 B 0.5744(10) 0.506(17) 0.7388(4) 0.042(4) Uani 0.582(6) 1 d D U P B 1

O3 O 0.6575(4) 0.5397(15) 0.7463(3) 0.057(2) Uani 0.582(6) 1 d D U P B 1

O4 O 0.5449(3) 0.4711(13) 0.8027(4) 0.0558(17) Uani 0.582(6) 1 d D U P B 1

C15 C 0.6813(4) 0.5702(11) 0.8233(4) 0.0580(15) Uani 0.582(6) 1 d D U P B 1

C16 C 0.6168(4) 0.4511(10) 0.8562(3) 0.0548(15) Uani 0.582(6) 1 d D U P B 1

C17 C 0.7679(4) 0.542(4) 0.8395(7) 0.075(4) Uani 0.582(6) 1 d D U P B 1

H17A H 0.7833 0.5616 0.8900 0.113 Uiso 0.582(6) 1 calc R U P B 1

H17B H 0.7815 0.4174 0.8273 0.113 Uiso 0.582(6) 1 calc R U P B 1

H17C H 0.7973 0.6265 0.8122 0.113 Uiso 0.582(6) 1 calc R U P B 1

C18 C 0.6699(8) 0.7708(15) 0.8357(6) 0.086(3) Uani 0.582(6) 1 d D U P B 1

H18A H 0.6125 0.8017 0.8258 0.128 Uiso 0.582(6) 1 calc R U P B 1

H18B H 0.6886 0.8001 0.8849 0.128 Uiso 0.582(6) 1 calc R U P B 1

H18C H 0.7013 0.8401 0.8043 0.128 Uiso 0.582(6) 1 calc R U P B 1

C19 C 0.6373(6) 0.2494(14) 0.8580(7) 0.073(3) Uani 0.582(6) 1 d D U P B 1

H19A H 0.6844 0.2278 0.8925 0.109 Uiso 0.582(6) 1 calc R U P B 1

H19B H 0.5909 0.1805 0.8714 0.109 Uiso 0.582(6) 1 calc R U P B 1

H19C H 0.6495 0.2106 0.8112 0.109 Uiso 0.582(6) 1 calc R U P B 1

C20 C 0.5938(8) 0.513(6) 0.9274(4) 0.071(3) Uani 0.582(6) 1 d D U P B 1

H20A H 0.6405 0.5002 0.9631 0.106 Uiso 0.582(6) 1 calc R U P B 1

H20B H 0.5768 0.6396 0.9244 0.106 Uiso 0.582(6) 1 calc R U P B 1

H20C H 0.5491 0.4381 0.9407 0.106 Uiso 0.582(6) 1 calc R U P B 1

B2' B 0.5743(14) 0.52(2) 0.7366(6) 0.042(4) Uani 0.418(6) 1 d D U P B 2

O3' O 0.6541(6) 0.4574(17) 0.7469(4) 0.057(2) Uani 0.418(6) 1 d D U P B 2

O4' O 0.5441(5) 0.5650(17) 0.7987(6) 0.0558(17) Uani 0.418(6) 1 d D U P B 2

C15' C 0.6790(5) 0.4502(12) 0.8241(5) 0.0580(15) Uani 0.418(6) 1 d D U P B 2

C16' C 0.6134(6) 0.5696(12) 0.8552(4) 0.0548(15) Uani 0.418(6) 1 d D U P B 2

C17' C 0.7649(6) 0.493(5) 0.8381(10) 0.075(4) Uani 0.418(6) 1 d D U P B 2

H17D H 0.7812 0.4886 0.8890 0.113 Uiso 0.418(6) 1 calc R U P B 2

H17E H 0.7965 0.4055 0.8141 0.113 Uiso 0.418(6) 1 calc R U P B 2

H17F H 0.7746 0.6151 0.8206 0.113 Uiso 0.418(6) 1 calc R U P B 2

C18' C 0.6734(9) 0.2530(18) 0.8449(9) 0.073(3) Uani 0.418(6) 1 d D U P B 2

H18D H 0.6169 0.2133 0.8370 0.109 Uiso 0.418(6) 1 calc R U P B 2

H18E H 0.7064 0.1795 0.8162 0.109 Uiso 0.418(6) 1 calc R U P B 2

H18F H 0.6932 0.2388 0.8949 0.109 Uiso 0.418(6) 1 calc R U P B 2

C19' C 0.6377(11) 0.7685(18) 0.8639(9) 0.086(3) Uani 0.418(6) 1 d D U P B 2

H19D H 0.6834 0.7796 0.9007 0.128 Uiso 0.418(6) 1 calc R U P B 2

H19E H 0.6534 0.8148 0.8192 0.128 Uiso 0.418(6) 1 calc R U P B 2

H19F H 0.5917 0.8386 0.8774 0.128 Uiso 0.418(6) 1 calc R U P B 2

C20' C 0.5847(12) 0.497(8) 0.9231(6) 0.071(3) Uani 0.418(6) 1 d D U P B 2

H20D H 0.6297 0.4992 0.9608 0.106 Uiso 0.418(6) 1 calc R U P B 2

H20E H 0.5405 0.5728 0.9365 0.106 Uiso 0.418(6) 1 calc R U P B 2

H20F H 0.5655 0.3726 0.9155 0.106 Uiso 0.418(6) 1 calc R U P B 2

C1S C 0.54647(19) 1.0139(12) 0.50572(17) 0.0414(7) Uani 1 1 d . . . . .

H1S1 H 0.5651 0.9068 0.5341 0.050 Uiso 1 1 calc R U . . .

H1S2 H 0.5651 1.1230 0.5328 0.050 Uiso 1 1 calc R U . . .

C2S C 0.5852(2) 1.0109(12) 0.43557(17) 0.0462(8) Uani 1 1 d . . . . .

H2S1 H 0.5664 0.9021 0.4083 0.055 Uiso 1 1 calc R U . . .

H2S2 H 0.5670 1.1184 0.4073 0.055 Uiso 1 1 calc R U . . .

C3S C 0.6782(2) 1.0097(14) 0.4479(2) 0.0572(10) Uani 1 1 d . . . . .

H3S1 H 0.6968 1.1108 0.4787 0.086 Uiso 1 1 calc R U . . .

H3S2 H 0.7003 1.0220 0.4026 0.086 Uiso 1 1 calc R U . . .

H3S3 H 0.6967 0.8956 0.4703 0.086 Uiso 1 1 calc R U . . .

loop_

_atom_site_aniso_label

_atom_site_aniso_U_11

_atom_site_aniso_U_22

_atom_site_aniso_U_33

_atom_site_aniso_U_23

_atom_site_aniso_U_13

_atom_site_aniso_U_12

C1 0.0290(13) 0.0432(16) 0.0170(11) -0.003(3) 0.0011(9) -0.003(3)

C2 0.0263(13) 0.0357(15) 0.0213(12) -0.006(2) -0.0009(10) 0.002(2)

C3 0.0241(12) 0.0319(14) 0.0214(12) -0.005(3) 0.0004(9) 0.001(2)

C4 0.0230(12) 0.0287(13) 0.0203(13) -0.003(3) -0.0011(10) 0.000(2)

C5 0.0256(12) 0.0312(14) 0.0194(12) 0.002(3) -0.0014(9) -0.004(2)

C6 0.0291(13) 0.0373(15) 0.0210(12) -0.003(3) 0.0011(10) 0.001(2)

C7 0.0230(12) 0.0432(16) 0.0228(12) -0.005(3) -0.0021(9) -0.001(2)

C8 0.0234(12) 0.0445(16) 0.0224(12) -0.003(3) -0.0035(9) 0.001(3)

B1 0.0319(16) 0.0469(19) 0.0257(14) -0.008(3) 0.0022(12) -0.004(3)

O1 0.0279(12) 0.066(4) 0.0278(11) -0.009(4) 0.0034(9) 0.003(3)

O2 0.0270(12) 0.060(2) 0.0291(12) -0.007(3) 0.0065(10) -0.007(3)

C9 0.0255(17) 0.067(4) 0.0378(19) -0.011(4) 0.0057(14) -0.003(4)

C10 0.0244(17) 0.066(3) 0.038(2) -0.015(4) 0.0086(15) -0.006(3)

C11 0.0447(19) 0.102(8) 0.0396(18) -0.017(4) 0.0134(15) -0.007(4)

C12 0.044(5) 0.063(4) 0.075(9) -0.024(5) 0.006(6) 0.000(3)

C13 0.049(4) 0.064(4) 0.064(9) -0.010(5) 0.019(6) -0.013(3)

C14 0.0356(17) 0.088(11) 0.047(2) -0.009(5) -0.0016(15) -0.001(4)

B1' 0.0319(16) 0.0469(19) 0.0257(14) -0.008(3) 0.0022(12) -0.004(3)

O1' 0.0279(12) 0.066(4) 0.0278(11) -0.009(4) 0.0034(9) 0.003(3)

O2' 0.0270(12) 0.060(2) 0.0291(12) -0.007(3) 0.0065(10) -0.007(3)

C9' 0.0255(17) 0.067(4) 0.0378(19) -0.011(4) 0.0057(14) -0.003(4)

C10' 0.0244(17) 0.066(3) 0.038(2) -0.015(4) 0.0086(15) -0.006(3)

C11' 0.0447(19) 0.102(8) 0.0396(18) -0.017(4) 0.0134(15) -0.007(4)

C12' 0.049(4) 0.064(4) 0.064(9) -0.010(5) 0.019(6) -0.013(3)

C13' 0.044(5) 0.063(4) 0.075(9) -0.024(5) 0.006(6) 0.000(3)

C14' 0.0356(17) 0.088(11) 0.047(2) -0.009(5) -0.0016(15) -0.001(4)

B2 0.0408(18) 0.055(12) 0.0315(17) -0.006(4) 0.0024(14) -0.020(3)

O3 0.0441(16) 0.098(6) 0.0269(12) -0.008(4) -0.0017(10) -0.041(4)

O4 0.0434(15) 0.091(5) 0.0317(15) -0.009(4) 0.0002(11) -0.036(4)

C15 0.049(2) 0.091(4) 0.0309(19) -0.002(4) -0.0064(16) -0.035(4)

C16 0.052(2) 0.084(4) 0.0259(18) 0.002(4) -0.0070(16) -0.031(4)

C17 0.052(2) 0.129(12) 0.042(2) -0.015(5) -0.0084(17) -0.033(4)

C18 0.105(9) 0.092(5) 0.057(6) -0.019(4) -0.004(5) -0.050(5)

C19 0.058(7) 0.097(5) 0.060(6) 0.014(4) -0.013(5) -0.024(5)

C20 0.058(3) 0.120(6) 0.0325(19) 0.002(4) -0.004(2) -0.030(6)

B2' 0.0408(18) 0.055(12) 0.0315(17) -0.006(4) 0.0024(14) -0.020(3)

O3' 0.0441(16) 0.098(6) 0.0269(12) -0.008(4) -0.0017(10) -0.041(4)

O4' 0.0434(15) 0.091(5) 0.0317(15) -0.009(4) 0.0002(11) -0.036(4)

C15' 0.049(2) 0.091(4) 0.0309(19) -0.002(4) -0.0064(16) -0.035(4)

C16' 0.052(2) 0.084(4) 0.0259(18) 0.002(4) -0.0070(16) -0.031(4)

C17' 0.052(2) 0.129(12) 0.042(2) -0.015(5) -0.0084(17) -0.033(4)

C18' 0.058(7) 0.097(5) 0.060(6) 0.014(4) -0.013(5) -0.024(5)

C19' 0.105(9) 0.092(5) 0.057(6) -0.019(4) -0.004(5) -0.050(5)

C20' 0.058(3) 0.120(6) 0.0325(19) 0.002(4) -0.004(2) -0.030(6)

C1S 0.0461(18) 0.0409(18) 0.0366(15) -0.005(3) 0.0015(12) -0.003(3)

C2S 0.0498(18) 0.051(2) 0.0382(16) 0.001(3) 0.0057(13) -0.003(3)

C3S 0.0492(19) 0.074(3) 0.0492(19) 0.011(4) 0.0100(15) -0.008(4)

_geom_special_details

;

All esds (except the esd in the dihedral angle between two l.s. planes)

are estimated using the full covariance matrix. The cell esds are taken

into account individually in the estimation of esds in distances, angles

and torsion angles; correlations between esds in cell parameters are only

used when they are defined by crystal symmetry. An approximate (isotropic)

treatment of cell esds is used for estimating esds involving l.s. planes.

;

loop_

_geom_bond_atom_site_label_1

_geom_bond_atom_site_label_2

_geom_bond_distance

_geom_bond_site_symmetry_2

_geom_bond_publ_flag

C1 C6 1.391(4) . ?

C1 C2 1.404(3) . ?

C1 H1 0.9400 . ?

C2 C3 1.410(3) . ?

C2 B1 1.564(4) . ?

C3 C4 1.426(4) . ?

C3 C7 1.438(3) . ?

C4 C5 1.427(3) . ?

C4 C4 1.435(5) 2_656 ?

C5 C6 1.412(3) . ?

C5 C8 1.434(4) 2_656 ?

C6 B2' 1.567(14) . ?

C6 B2 1.600(7) . ?

C7 C8 1.343(4) . ?

C7 H7 0.9400 . ?

C8 C5 1.434(4) 2_656 ?

C8 H8 0.9400 . ?

B1 O1 1.365(5) . ?

B1 O2 1.387(5) . ?

O1 C9 1.473(5) . ?

O2 C10 1.454(5) . ?

C9 C11 1.489(17) . ?

C9 C12 1.539(13) . ?

C9 C10 1.542(6) . ?

C10 C14 1.496(10) . ?

C10 C13 1.527(14) . ?

C11 H11A 0.9700 . ?

C11 H11B 0.9700 . ?

C11 H11C 0.9700 . ?

C12 H12A 0.9700 . ?

C12 H12B 0.9700 . ?

C12 H12C 0.9700 . ?

C13 H13A 0.9700 . ?

C13 H13B 0.9700 . ?

C13 H13C 0.9700 . ?

C14 H14A 0.9700 . ?

C14 H14B 0.9700 . ?

C14 H14C 0.9700 . ?

O1' C9' 1.472(5) . ?

O2' C10' 1.453(5) . ?

C9' C11' 1.490(17) . ?

C9' C12' 1.540(13) . ?

C9' C10' 1.541(7) . ?

C10' C14' 1.497(10) . ?

C10' C13' 1.527(14) . ?

C11' H11D 0.9700 . ?

C11' H11E 0.9700 . ?

C11' H11F 0.9700 . ?

C12' H12D 0.9700 . ?

C12' H12E 0.9700 . ?

C12' H12F 0.9700 . ?

C13' H13D 0.9700 . ?

C13' H13E 0.9700 . ?

C13' H13F 0.9700 . ?

C14' H14D 0.9700 . ?

C14' H14E 0.9700 . ?

C14' H14F 0.9700 . ?

B2 O4 1.37(3) . ?

B2 O3 1.38(4) . ?

O3 C15 1.479(5) . ?

O4 C16 1.477(5) . ?

C15 C17 1.441(9) . ?

C15 C18 1.503(11) . ?

C15 C16 1.555(7) . ?

C16 C20 1.504(17) . ?

C16 C19 1.515(11) . ?

C17 H17A 0.9700 . ?

C17 H17B 0.9700 . ?

C17 H17C 0.9700 . ?

C18 H18A 0.9700 . ?

C18 H18B 0.9700 . ?

C18 H18C 0.9700 . ?

C19 H19A 0.9700 . ?

C19 H19B 0.9700 . ?

C19 H19C 0.9700 . ?

C20 H20A 0.9700 . ?

C20 H20B 0.9700 . ?

C20 H20C 0.9700 . ?

B2' O4' 1.37(3) . ?

B2' O3' 1.38(4) . ?

O3' C15' 1.471(6) . ?

O4' C16' 1.473(6) . ?

C15' C17' 1.444(9) . ?

C15' C18' 1.502(11) . ?

C15' C16' 1.553(8) . ?

C16' C20' 1.509(17) . ?

C16' C19' 1.515(11) . ?

C17' H17D 0.9700 . ?

C17' H17E 0.9700 . ?

C17' H17F 0.9700 . ?

C18' H18D 0.9700 . ?

C18' H18E 0.9700 . ?

C18' H18F 0.9700 . ?

C19' H19D 0.9700 . ?

C19' H19E 0.9700 . ?

C19' H19F 0.9700 . ?

C20' H20D 0.9700 . ?

C20' H20E 0.9700 . ?

C20' H20F 0.9700 . ?

C1S C1S 1.521(6) 2_656 ?

C1S C2S 1.530(5) . ?

C1S H1S1 0.9800 . ?

C1S H1S2 0.9800 . ?

C2S C3S 1.523(5) . ?

C2S H2S1 0.9800 . ?

C2S H2S2 0.9800 . ?

C3S H3S1 0.9700 . ?

C3S H3S2 0.9700 . ?

C3S H3S3 0.9700 . ?

loop_

_geom_angle_atom_site_label_1

_geom_angle_atom_site_label_2

_geom_angle_atom_site_label_3

_geom_angle

_geom_angle_site_symmetry_1

_geom_angle_site_symmetry_3

_geom_angle_publ_flag

C6 C1 C2 123.2(2) . . ?

C6 C1 H1 118.4 . . ?

C2 C1 H1 118.4 . . ?

C1 C2 C3 118.4(2) . . ?

C1 C2 B1 117.0(2) . . ?

C3 C2 B1 124.5(2) . . ?

C2 C3 C4 120.0(2) . . ?

C2 C3 C7 122.3(2) . . ?

C4 C3 C7 117.8(2) . . ?

C3 C4 C5 119.8(2) . . ?

C3 C4 C4 120.1(3) . 2_656 ?

C5 C4 C4 120.1(3) . 2_656 ?

C6 C5 C4 119.8(2) . . ?

C6 C5 C8 122.2(2) . 2_656 ?

C4 C5 C8 117.9(2) . 2_656 ?

C1 C6 C5 118.8(2) . . ?

C1 C6 B2' 117.5(9) . . ?

C5 C6 B2' 123.8(11) . . ?

C1 C6 B2 116.6(8) . . ?

C5 C6 B2 124.6(6) . . ?

C8 C7 C3 122.1(2) . . ?

C8 C7 H7 119.0 . . ?

C3 C7 H7 119.0 . . ?

C7 C8 C5 122.0(2) . 2_656 ?

C7 C8 H8 119.0 . . ?

C5 C8 H8 119.0 2_656 . ?

O1 B1 O2 112.6(4) . . ?

O1 B1 C2 121.8(3) . . ?

O2 B1 C2 125.5(3) . . ?

B1 O1 C9 106.8(4) . . ?

B1 O2 C10 106.7(4) . . ?

O1 C9 C11 109.6(7) . . ?

O1 C9 C12 105.7(8) . . ?

C11 C9 C12 109(3) . . ?

O1 C9 C10 101.9(4) . . ?

C11 C9 C10 118(2) . . ?

C12 C9 C10 111.9(6) . . ?

O2 C10 C14 108.4(6) . . ?

O2 C10 C13 106.1(7) . . ?

C14 C10 C13 109.7(13) . . ?

O2 C10 C9 103.0(4) . . ?

C14 C10 C9 116.8(11) . . ?

C13 C10 C9 112.1(6) . . ?

C9 C11 H11A 109.5 . . ?

C9 C11 H11B 109.5 . . ?

H11A C11 H11B 109.5 . . ?

C9 C11 H11C 109.5 . . ?

H11A C11 H11C 109.5 . . ?

H11B C11 H11C 109.5 . . ?

C9 C12 H12A 109.5 . . ?

C9 C12 H12B 109.5 . . ?

H12A C12 H12B 109.5 . . ?

C9 C12 H12C 109.5 . . ?

H12A C12 H12C 109.5 . . ?

H12B C12 H12C 109.5 . . ?

C10 C13 H13A 109.5 . . ?

C10 C13 H13B 109.5 . . ?

H13A C13 H13B 109.5 . . ?

C10 C13 H13C 109.5 . . ?

H13A C13 H13C 109.5 . . ?

H13B C13 H13C 109.5 . . ?

C10 C14 H14A 109.5 . . ?

C10 C14 H14B 109.5 . . ?

H14A C14 H14B 109.5 . . ?

C10 C14 H14C 109.5 . . ?

H14A C14 H14C 109.5 . . ?

H14B C14 H14C 109.5 . . ?

O1' C9' C11' 109.6(7) . . ?

O1' C9' C12' 105.1(8) . . ?

C11' C9' C12' 110(3) . . ?

O1' C9' C10' 102.3(4) . . ?

C11' C9' C10' 117(2) . . ?

C12' C9' C10' 111.9(7) . . ?

O2' C10' C14' 108.4(6) . . ?

O2' C10' C13' 106.3(8) . . ?

C14' C10' C13' 109.3(13) . . ?

O2' C10' C9' 103.1(4) . . ?

C14' C10' C9' 116.4(11) . . ?

C13' C10' C9' 112.6(6) . . ?

C9' C11' H11D 109.5 . . ?

C9' C11' H11E 109.5 . . ?

H11D C11' H11E 109.5 . . ?

C9' C11' H11F 109.5 . . ?

H11D C11' H11F 109.5 . . ?

H11E C11' H11F 109.5 . . ?

C9' C12' H12D 109.5 . . ?

C9' C12' H12E 109.5 . . ?

H12D C12' H12E 109.5 . . ?

C9' C12' H12F 109.5 . . ?

H12D C12' H12F 109.5 . . ?

H12E C12' H12F 109.5 . . ?

C10' C13' H13D 109.5 . . ?

C10' C13' H13E 109.5 . . ?

H13D C13' H13E 109.5 . . ?

C10' C13' H13F 109.5 . . ?

H13D C13' H13F 109.5 . . ?

H13E C13' H13F 109.5 . . ?

C10' C14' H14D 109.5 . . ?

C10' C14' H14E 109.5 . . ?

H14D C14' H14E 109.5 . . ?

C10' C14' H14F 109.5 . . ?

H14D C14' H14F 109.5 . . ?

H14E C14' H14F 109.5 . . ?

O4 B2 O3 112.6(4) . . ?

O4 B2 C6 122(2) . . ?

O3 B2 C6 125(3) . . ?

B2 O3 C15 106.2(5) . . ?

B2 O4 C16 106.7(4) . . ?

C17 C15 O3 109.7(6) . . ?

C17 C15 C18 104.2(12) . . ?

O3 C15 C18 106.0(6) . . ?

C17 C15 C16 122.5(10) . . ?

O3 C15 C16 100.9(4) . . ?

C18 C15 C16 112.6(6) . . ?

O4 C16 C20 108.7(6) . . ?

O4 C16 C19 105.7(6) . . ?

C20 C16 C19 110.5(18) . . ?

O4 C16 C15 101.6(4) . . ?

C20 C16 C15 116.0(15) . . ?

C19 C16 C15 113.3(6) . . ?

C15 C17 H17A 109.5 . . ?

C15 C17 H17B 109.5 . . ?

H17A C17 H17B 109.5 . . ?

C15 C17 H17C 109.5 . . ?

H17A C17 H17C 109.5 . . ?

H17B C17 H17C 109.5 . . ?

C15 C18 H18A 109.5 . . ?

C15 C18 H18B 109.5 . . ?

H18A C18 H18B 109.5 . . ?

C15 C18 H18C 109.5 . . ?

H18A C18 H18C 109.5 . . ?

H18B C18 H18C 109.5 . . ?

C16 C19 H19A 109.5 . . ?

C16 C19 H19B 109.5 . . ?

H19A C19 H19B 109.5 . . ?

C16 C19 H19C 109.5 . . ?

H19A C19 H19C 109.5 . . ?

H19B C19 H19C 109.5 . . ?

C16 C20 H20A 109.5 . . ?

C16 C20 H20B 109.5 . . ?

H20A C20 H20B 109.5 . . ?

C16 C20 H20C 109.5 . . ?

H20A C20 H20C 109.5 . . ?

H20B C20 H20C 109.5 . . ?

O4' B2' O3' 113.0(7) . . ?

O4' B2' C6 119.9(16) . . ?

O3' B2' C6 127(4) . . ?

B2' O3' C15' 108.0(5) . . ?

B2' O4' C16' 107.5(5) . . ?

C17' C15' O3' 109.5(7) . . ?

C17' C15' C18' 104.5(13) . . ?

O3' C15' C18' 105.9(7) . . ?

C17' C15' C16' 121.0(12) . . ?

O3' C15' C16' 102.9(5) . . ?

C18' C15' C16' 112.1(7) . . ?

O4' C16' C20' 108.5(8) . . ?

O4' C16' C19' 106.0(7) . . ?

C20' C16' C19' 110.4(19) . . ?

O4' C16' C15' 103.3(5) . . ?

C20' C16' C15' 114.6(16) . . ?

C19' C16' C15' 113.3(7) . . ?

C15' C17' H17D 109.5 . . ?

C15' C17' H17E 109.5 . . ?

H17D C17' H17E 109.5 . . ?

C15' C17' H17F 109.5 . . ?

H17D C17' H17F 109.5 . . ?

H17E C17' H17F 109.5 . . ?

C15' C18' H18D 109.5 . . ?

C15' C18' H18E 109.5 . . ?

H18D C18' H18E 109.5 . . ?

C15' C18' H18F 109.5 . . ?

H18D C18' H18F 109.5 . . ?

H18E C18' H18F 109.5 . . ?

C16' C19' H19D 109.5 . . ?

C16' C19' H19E 109.5 . . ?

H19D C19' H19E 109.5 . . ?

C16' C19' H19F 109.5 . . ?

H19D C19' H19F 109.5 . . ?

H19E C19' H19F 109.5 . . ?

C16' C20' H20D 109.5 . . ?

C16' C20' H20E 109.5 . . ?

H20D C20' H20E 109.5 . . ?

C16' C20' H20F 109.5 . . ?

H20D C20' H20F 109.5 . . ?

H20E C20' H20F 109.5 . . ?

C1S C1S C2S 112.6(3) 2_656 . ?

C1S C1S H1S1 109.1 2_656 . ?

C2S C1S H1S1 109.1 . . ?

C1S C1S H1S2 109.1 2_656 . ?

C2S C1S H1S2 109.1 . . ?

H1S1 C1S H1S2 107.8 . . ?

C3S C2S C1S 112.0(3) . . ?

C3S C2S H2S1 109.2 . . ?

C1S C2S H2S1 109.2 . . ?

C3S C2S H2S2 109.2 . . ?

C1S C2S H2S2 109.2 . . ?

H2S1 C2S H2S2 107.9 . . ?

C2S C3S H3S1 109.5 . . ?

C2S C3S H3S2 109.5 . . ?

H3S1 C3S H3S2 109.5 . . ?

C2S C3S H3S3 109.5 . . ?

H3S1 C3S H3S3 109.5 . . ?

H3S2 C3S H3S3 109.5 . . ?

loop_

_geom_torsion_atom_site_label_1

_geom_torsion_atom_site_label_2

_geom_torsion_atom_site_label_3

_geom_torsion_atom_site_label_4

_geom_torsion

_geom_torsion_site_symmetry_1

_geom_torsion_site_symmetry_2

_geom_torsion_site_symmetry_3

_geom_torsion_site_symmetry_4

_geom_torsion_publ_flag

C6 C1 C2 C3 2.5(10) . . . . ?

C6 C1 C2 B1 -179.0(6) . . . . ?

C1 C2 C3 C4 -1.5(9) . . . . ?

B1 C2 C3 C4 -179.8(5) . . . . ?

C1 C2 C3 C7 178.3(6) . . . . ?

B1 C2 C3 C7 0.0(11) . . . . ?

C2 C3 C4 C5 0.6(8) . . . . ?

C7 C3 C4 C5 -179.2(6) . . . . ?

C2 C3 C4 C4 -179.9(4) . . . 2_656 ?

C7 C3 C4 C4 0.3(6) . . . 2_656 ?

C3 C4 C5 C6 -0.5(8) . . . . ?

C4 C4 C5 C6 179.9(4) 2_656 . . . ?

C3 C4 C5 C8 179.6(6) . . . 2_656 ?

C4 C4 C5 C8 0.0(7) 2_656 . . 2_656 ?

C2 C1 C6 C5 -2.5(10) . . . . ?

C2 C1 C6 B2' 177(7) . . . . ?

C2 C1 C6 B2 180(5) . . . . ?

C4 C5 C6 C1 1.4(9) . . . . ?

C8 C5 C6 C1 -178.7(6) 2_656 . . . ?

C4 C5 C6 B2' -178(8) . . . . ?

C8 C5 C6 B2' 2(8) 2_656 . . . ?

C4 C5 C6 B2 179(6) . . . . ?

C8 C5 C6 B2 -1(6) 2_656 . . . ?

C2 C3 C7 C8 -179.7(6) . . . . ?

C4 C3 C7 C8 0.1(10) . . . . ?

C3 C7 C8 C5 -0.4(11) . . . 2_656 ?

C1 C2 B1 O1 17.7(11) . . . . ?

C3 C2 B1 O1 -164.0(7) . . . . ?

C1 C2 B1 O2 -157.8(7) . . . . ?

C3 C2 B1 O2 20.5(11) . . . . ?

O2 B1 O1 C9 -12.0(9) . . . . ?

C2 B1 O1 C9 172.0(6) . . . . ?

O1 B1 O2 C10 -8.1(9) . . . . ?

C2 B1 O2 C10 167.8(6) . . . . ?

B1 O1 C9 C11 151(3) . . . . ?

B1 O1 C9 C12 -91.6(9) . . . . ?

B1 O1 C9 C10 25.4(8) . . . . ?

B1 O2 C10 C14 147.8(13) . . . . ?

B1 O2 C10 C13 -94.5(8) . . . . ?

B1 O2 C10 C9 23.4(7) . . . . ?

O1 C9 C10 O2 -29.4(7) . . . . ?

C11 C9 C10 O2 -149.2(17) . . . . ?

C12 C9 C10 O2 83.1(9) . . . . ?

O1 C9 C10 C14 -148.1(10) . . . . ?

C11 C9 C10 C14 92.1(19) . . . . ?

C12 C9 C10 C14 -35.6(13) . . . . ?

O1 C9 C10 C13 84.2(10) . . . . ?

C11 C9 C10 C13 -35.6(19) . . . . ?

C12 C9 C10 C13 -163.3(8) . . . . ?

O1' C9' C10' O2' 26.9(7) . . . . ?

C11' C9' C10' O2' 147.0(19) . . . . ?

C12' C9' C10' O2' -85.1(10) . . . . ?

O1' C9' C10' C14' 145.4(11) . . . . ?

C11' C9' C10' C14' -95(2) . . . . ?

C12' C9' C10' C14' 33.4(14) . . . . ?

O1' C9' C10' C13' -87.2(10) . . . . ?

C11' C9' C10' C13' 33(2) . . . . ?

C12' C9' C10' C13' 160.8(9) . . . . ?

C1 C6 B2 O4 11(11) . . . . ?

C5 C6 B2 O4 -167(6) . . . . ?

B2' C6 B2 O4 119(49) . . . . ?

C1 C6 B2 O3 -168(7) . . . . ?

C5 C6 B2 O3 14(12) . . . . ?

B2' C6 B2 O3 -60(32) . . . . ?

O4 B2 O3 C15 -14(9) . . . . ?

C6 B2 O3 C15 165(7) . . . . ?

O3 B2 O4 C16 -9(9) . . . . ?

C6 B2 O4 C16 172(7) . . . . ?

B2 O3 C15 C17 160(5) . . . . ?

B2 O3 C15 C18 -88(5) . . . . ?

B2 O3 C15 C16 29(5) . . . . ?

B2 O4 C16 C20 150(6) . . . . ?

B2 O4 C16 C19 -92(5) . . . . ?

B2 O4 C16 C15 27(5) . . . . ?

C17 C15 C16 O4 -155.5(10) . . . . ?

O3 C15 C16 O4 -33.6(7) . . . . ?

C18 C15 C16 O4 79.1(7) . . . . ?

C17 C15 C16 C20 86.8(16) . . . . ?

O3 C15 C16 C20 -151.3(13) . . . . ?

C18 C15 C16 C20 -38.6(14) . . . . ?

C17 C15 C16 C19 -42.5(11) . . . . ?

O3 C15 C16 C19 79.4(8) . . . . ?

C18 C15 C16 C19 -168.0(7) . . . . ?

C1 C6 B2' O4' -16(16) . . . . ?

C5 C6 B2' O4' 163(8) . . . . ?

B2 C6 B2' O4' -89(30) . . . . ?

C1 C6 B2' O3' 155(10) . . . . ?

C5 C6 B2' O3' -26(18) . . . . ?

B2 C6 B2' O3' 82(54) . . . . ?

O4' B2' O3' C15' 5(14) . . . . ?

C6 B2' O3' C15' -167(11) . . . . ?

O3' B2' O4' C16' 11(13) . . . . ?

C6 B2' O4' C16' -177(10) . . . . ?

B2' O3' C15' C17' -147(8) . . . . ?

B2' O3' C15' C18' 101(8) . . . . ?

B2' O3' C15' C16' -17(8) . . . . ?

B2' O4' C16' C20' -143(8) . . . . ?

B2' O4' C16' C19' 99(8) . . . . ?

B2' O4' C16' C15' -21(8) . . . . ?

C17' C15' C16' O4' 145.0(13) . . . . ?

O3' C15' C16' O4' 22.5(8) . . . . ?

C18' C15' C16' O4' -91.0(9) . . . . ?

C17' C15' C16' C20' -97.2(19) . . . . ?

O3' C15' C16' C20' 140.3(15) . . . . ?

C18' C15' C16' C20' 26.9(17) . . . . ?

C17' C15' C16' C19' 30.8(15) . . . . ?

O3' C15' C16' C19' -91.7(9) . . . . ?

C18' C15' C16' C19' 154.9(9) . . . . ?

C1S C1S C2S C3S -179.6(5) 2_656 . . . ?

_refine_diff_density_max 0.348

_refine_diff_density_min -0.491

_refine_diff_density_rms 0.059

_shelxl_version_number 2014-3

_iucr_refine_instructions_details

;

TITL x14361 in I2

CELL 1.54178 16.4584 7.3229 18.8669 90.000 96.268 90.000

ZERR 2.00 0.00040 0.00020 0.00072 0.0000 0.0011 0.0000

LATT -2

SYMM -X, Y, -Z

SFAC C H B O

UNIT 92 136 8 16

TEMP -63.000

SIZE 0.25 0.20 0.02

REM COLR colourless

REM FORM shard

REM Many reflections were split into three at 90K. These split reflections

REM eventually coalesce as the temperature rises, and were single at about

REM 200K. On further warming to around 230K, the diffraction pattern seemed

REM to get a little fuzzier. For this reason data were collected with the

REM crystal at 210K.

REM

REM Structure was refined as twinned across a mirror plane perpendicular to

REM the b-axis. This is entirely equivalent (in this case) to being twinned

REM by inversion (owing the the 2-fold).

BOND $H

TWIN 1 0 0 0 -1 0 0 0 1

L.S. 24

CONF

LIST 4

ACTA

FMAP 2

PLAN 10

WGHT 0.129800 1.678200

FVAR 0.09154 0.52795 0.58237

C1 1 0.432333 0.507863 0.670003 11.00000 0.02905 0.04319 =

0.01699 -0.00340 0.00113 -0.00333

AFIX 43

H1 2 0.414215 0.503159 0.715524 11.00000 -1.20000

AFIX 0

C2 1 0.373600 0.511819 0.610200 11.00000 0.02629 0.03568 =

0.02129 -0.00650 -0.00095 0.00208

C3 1 0.400717 0.511424 0.541866 11.00000 0.02413 0.03191 =

0.02144 -0.00461 0.00035 0.00135

C4 1 0.486095 0.511548 0.534753 11.00000 0.02300 0.02868 =

0.02031 -0.00290 -0.00105 0.00008

C5 1 0.543781 0.510340 0.596949 11.00000 0.02558 0.03122 =

0.01941 0.00211 -0.00143 -0.00376

C6 1 0.516044 0.510567 0.665159 11.00000 0.02911 0.03726 =

0.02097 -0.00295 0.00109 0.00134

C7 1 0.344934 0.510301 0.477666 11.00000 0.02304 0.04319 =

0.02283 -0.00481 -0.00207 -0.00085

AFIX 43

H7 2 0.288429 0.510316 0.481319 11.00000 -1.20000

AFIX 0

C8 1 0.370990 0.509230 0.412636 11.00000 0.02339 0.04446 =

0.02244 -0.00350 -0.00350 0.00070

AFIX 43

H8 2 0.332189 0.507709 0.372199 11.00000 -1.20000

AFIX 0

EXYZ B1 B1'

EADP B1 B1'

EADP O1 O1'

EADP O2 O2'

EADP C9 C9'

EADP C10 C10'

EADP C11 C11'

EADP C12 C13'

EADP C13 C12'

EADP C14 C14'

SAME 0.005 B1' O1' O2' C9' C10' C11' C12' C13' C14'

RIGU 0.005 B1 > C14

RIGU 0.005 B1' > C14'

PART 1

B1 3 0.281781 0.511185 0.624534 21.00000 0.03192 0.04686 =

0.02573 -0.00831 0.00219 -0.00427

O1 4 0.258500 0.555388 0.689626 21.00000 0.02794 0.06568 =

0.02778 -0.00873 0.00344 0.00287

O2 4 0.216622 0.456153 0.576440 21.00000 0.02701 0.06016 =

0.02915 -0.00748 0.00645 -0.00730

C9 1 0.168572 0.566345 0.680848 21.00000 0.02547 0.06729 =

0.03783 -0.01053 0.00573 -0.00347

C10 1 0.146244 0.439816 0.616343 21.00000 0.02437 0.06633 =

0.03845 -0.01542 0.00859 -0.00635

C11 1 0.136637 0.517910 0.749294 21.00000 0.04471 0.10193 =

0.03962 -0.01681 0.01337 -0.00666

AFIX 33

H11A 2 0.077436 0.525181 0.743436 21.00000 -1.50000

H11B 2 0.153337 0.394594 0.762830 21.00000 -1.50000

H11C 2 0.158338 0.602407 0.786197 21.00000 -1.50000

AFIX 0

C12 1 0.148050 0.767259 0.662836 21.00000 0.04417 0.06340 =

0.07544 -0.02391 0.00588 -0.00030

AFIX 33

H12A 2 0.089187 0.783002 0.656398 21.00000 -1.50000

H12B 2 0.171423 0.844707 0.701516 21.00000 -1.50000

H12C 2 0.170608 0.800685 0.619242 21.00000 -1.50000

AFIX 0

C13 1 0.142598 0.239577 0.638305 21.00000 0.04854 0.06401 =

0.06444 -0.01020 0.01924 -0.01261

AFIX 33

H13A 2 0.096379 0.221019 0.665291 21.00000 -1.50000

H13B 2 0.136423 0.163327 0.596019 21.00000 -1.50000

H13C 2 0.192673 0.207162 0.667559 21.00000 -1.50000

AFIX 0

C14 1 0.071163 0.487639 0.567885 21.00000 0.03558 0.08827 =

0.04702 -0.00927 -0.00164 -0.00105

AFIX 33

H14A 2 0.023553 0.477409 0.593733 21.00000 -1.50000

H14B 2 0.075669 0.611886 0.550925 21.00000 -1.50000

H14C 2 0.065595 0.404624 0.527583 21.00000 -1.50000

AFIX 0

PART 2

B1' 3 0.281781 0.511185 0.624534 -21.00000 0.03192 0.04686 =

0.02573 -0.00831 0.00219 -0.00427

O1' 4 0.257923 0.468466 0.689538 -21.00000 0.02794 0.06568 =

0.02778 -0.00873 0.00344 0.00287

O2' 4 0.217617 0.575967 0.577554 -21.00000 0.02701 0.06016 =

0.02915 -0.00748 0.00645 -0.00730

C9' 1 0.168128 0.456764 0.679962 -21.00000 0.02547 0.06729 =

0.03783 -0.01053 0.00573 -0.00347

C10' 1 0.145614 0.583630 0.615656 -21.00000 0.02437 0.06633 =

0.03845 -0.01542 0.00859 -0.00635

C11' 1 0.135197 0.504196 0.748090 -21.00000 0.04471 0.10193 =

0.03962 -0.01681 0.01337 -0.00666

AFIX 33

H11D 2 0.076016 0.496396 0.741659 -21.00000 -1.50000

H11E 2 0.156609 0.419541 0.785043 -21.00000 -1.50000

H11F 2 0.151521 0.627538 0.761935 -21.00000 -1.50000

AFIX 0

C12' 1 0.149131 0.255388 0.661452 -21.00000 0.04854 0.06401 =

0.06444 -0.01020 0.01924 -0.01261

AFIX 33

H12D 2 0.090392 0.237770 0.654362 -21.00000 -1.50000

H12E 2 0.172549 0.223347 0.618116 -21.00000 -1.50000

H12F 2 0.172483 0.178178 0.700216 -21.00000 -1.50000

AFIX 0

C13' 1 0.136493 0.782734 0.637590 -21.00000 0.04417 0.06340 =

0.07544 -0.02391 0.00588 -0.00030

AFIX 33

H13D 2 0.088933 0.795063 0.663385 -21.00000 -1.50000

H13E 2 0.184940 0.820483 0.667969 -21.00000 -1.50000

H13F 2 0.129813 0.859095 0.595345 -21.00000 -1.50000

AFIX 0

C14' 1 0.072929 0.529231 0.565394 -21.00000 0.03558 0.08827 =

0.04702 -0.00927 -0.00164 -0.00105

AFIX 33

H14D 2 0.024203 0.533590 0.589997 -21.00000 -1.50000

H14E 2 0.066897 0.612747 0.525257 -21.00000 -1.50000

H14F 2 0.080594 0.406112 0.548362 -21.00000 -1.50000

AFIX 0

EADP B2 B2'

EADP O3 O3'

EADP O4 O4'

EADP C15 C15'

EADP C16 C16'

EADP C17 C17'

EADP C18 C19'

EADP C19 C18'

EADP C20 C20'

SAME 0.005 B2' O3' O4' C15' C16' C17' C18' C19' c20'

RIGU 0.005 B2 > C20

RIGU 0.005 B2' > C20'

ISOR B2 B2'

PART 1

B2 3 0.574368 0.506169 0.738788 31.00000 0.04075 0.05459 =

0.03149 -0.00615 0.00245 -0.02035

O3 4 0.657524 0.539719 0.746294 31.00000 0.04415 0.09783 =

0.02687 -0.00817 -0.00166 -0.04057

O4 4 0.544923 0.471130 0.802671 31.00000 0.04340 0.09143 =

0.03169 -0.00853 0.00020 -0.03598

C15 1 0.681262 0.570150 0.823271 31.00000 0.04943 0.09126 =

0.03090 -0.00159 -0.00641 -0.03501

C16 1 0.616777 0.451130 0.856236 31.00000 0.05200 0.08389 =

0.02593 0.00189 -0.00698 -0.03116

C17 1 0.767914 0.541600 0.839523 31.00000 0.05250 0.12925 =

0.04169 -0.01473 -0.00835 -0.03297

AFIX 33

H17A 2 0.783305 0.561644 0.890025 31.00000 -1.50000

H17B 2 0.781465 0.417440 0.827293 31.00000 -1.50000

H17C 2 0.797305 0.626498 0.812190 31.00000 -1.50000

AFIX 0

C18 1 0.669928 0.770787 0.835673 31.00000 0.10481 0.09238 =

0.05674 -0.01946 -0.00367 -0.04975

AFIX 33

H18A 2 0.612501 0.801720 0.825790 31.00000 -1.50000

H18B 2 0.688642 0.800058 0.884899 31.00000 -1.50000

H18C 2 0.701323 0.840105 0.804338 31.00000 -1.50000

AFIX 0

C19 1 0.637251 0.249368 0.857996 31.00000 0.05793 0.09682 =

0.05955 0.01389 -0.01293 -0.02417

AFIX 33

H19A 2 0.684373 0.227773 0.892540 31.00000 -1.50000

H19B 2 0.590949 0.180532 0.871404 31.00000 -1.50000

H19C 2 0.649539 0.210617 0.811171 31.00000 -1.50000

AFIX 0

C20 1 0.593766 0.512744 0.927448 31.00000 0.05799 0.12013 =

0.03248 0.00153 -0.00376 -0.02980

AFIX 33

H20A 2 0.640536 0.500233 0.963126 31.00000 -1.50000

H20B 2 0.576779 0.639599 0.924401 31.00000 -1.50000

H20C 2 0.549145 0.438127 0.940732 31.00000 -1.50000

AFIX 0

PART 2

B2' 3 0.574321 0.517039 0.736603 -31.00000 0.04075 0.05459 =

0.03149 -0.00615 0.00245 -0.02035

O3' 4 0.654105 0.457424 0.746862 -31.00000 0.04415 0.09783 =

0.02687 -0.00817 -0.00166 -0.04057

O4' 4 0.544104 0.564963 0.798694 -31.00000 0.04340 0.09143 =

0.03169 -0.00853 0.00020 -0.03598

C15' 1 0.679014 0.450206 0.824116 -31.00000 0.04943 0.09126 =

0.03090 -0.00159 -0.00641 -0.03501

C16' 1 0.613360 0.569555 0.855157 -31.00000 0.05200 0.08389 =

0.02593 0.00189 -0.00698 -0.03116

C17' 1 0.764892 0.493417 0.838107 -31.00000 0.05250 0.12925 =

0.04169 -0.01473 -0.00835 -0.03297

AFIX 33

H17D 2 0.781249 0.488620 0.889048 -31.00000 -1.50000

H17E 2 0.796498 0.405545 0.814118 -31.00000 -1.50000

H17F 2 0.774605 0.615105 0.820552 -31.00000 -1.50000

AFIX 0

C18' 1 0.673352 0.253019 0.844900 -31.00000 0.05793 0.09682 =

0.05955 0.01389 -0.01293 -0.02417

AFIX 33

H18D 2 0.616860 0.213350 0.836952 -31.00000 -1.50000

H18E 2 0.706354 0.179543 0.816246 -31.00000 -1.50000

H18F 2 0.693186 0.238805 0.894924 -31.00000 -1.50000

AFIX 0

C19' 1 0.637653 0.768547 0.863918 -31.00000 0.10481 0.09238 =

0.05674 -0.01946 -0.00367 -0.04975

AFIX 33

H19D 2 0.683363 0.779629 0.900733 -31.00000 -1.50000

H19E 2 0.653406 0.814799 0.819218 -31.00000 -1.50000

H19F 2 0.591742 0.838596 0.877410 -31.00000 -1.50000

AFIX 0

C20' 1 0.584698 0.497085 0.923059 -31.00000 0.05799 0.12013 =

0.03248 0.00153 -0.00376 -0.02980

AFIX 33

H20D 2 0.629694 0.499240 0.960814 -31.00000 -1.50000

H20E 2 0.540495 0.572797 0.936472 -31.00000 -1.50000

H20F 2 0.565481 0.372635 0.915537 -31.00000 -1.50000

AFIX 0

PART 0

REM n-hexane solvent

C1S 1 0.546472 1.013874 0.505718 11.00000 0.04605 0.04094 =

0.03662 -0.00472 0.00146 -0.00347

AFIX 23

H1S1 2 0.565072 0.906763 0.534127 11.00000 -1.20000

H1S2 2 0.565119 1.123035 0.532821 11.00000 -1.20000

AFIX 0

C2S 1 0.585177 1.010862 0.435572 11.00000 0.04983 0.05077 =

0.03821 0.00141 0.00571 -0.00317

AFIX 23

H2S1 2 0.566433 0.902052 0.408287 11.00000 -1.20000

H2S2 2 0.567034 1.118438 0.407251 11.00000 -1.20000

AFIX 0

C3S 1 0.678182 1.009688 0.447896 11.00000 0.04922 0.07420 =

0.04919 0.01063 0.00998 -0.00835

AFIX 137

H3S1 2 0.696780 1.110763 0.478722 11.00000 -1.50000

H3S2 2 0.700347 1.022000 0.402562 11.00000 -1.50000

H3S3 2 0.696655 0.895555 0.470253 11.00000 -1.50000

AFIX 0

HKLF 4

REM x14361 in I2

REM R1 = 0.0637 for 3212 Fo > 4sig(Fo) and 0.0709 for all 3702 data

REM 316 parameters refined using 349 restraints

END

WGHT 0.1299 1.6742

REM Highest difference peak 0.348, deepest hole -0.491, 1-sigma level 0.059

Q1 1 0.5180 0.5109 0.5716 11.00000 0.05 0.35

Q2 1 0.4989 0.5090 0.5491 11.00000 0.05 0.33

Q3 1 0.4405 0.5127 0.5387 11.00000 0.05 0.31

Q4 1 0.3929 0.5119 0.6326 11.00000 0.05 0.31

Q5 1 0.3469 0.5160 0.6144 11.00000 0.05 0.28

Q6 1 0.5394 0.5112 0.6895 11.00000 0.05 0.28

Q7 1 0.5725 0.5073 0.5918 11.00000 0.05 0.27

Q8 1 0.3639 0.5113 0.5002 11.00000 0.05 0.27

Q9 1 0.5324 0.5162 0.6342 11.00000 0.05 0.26

Q10 1 0.5070 1.0120 0.3961 11.00000 0.05 0.24

;

_shelx_res_checksum 12056
